# Coatomer protein complex I is required for efficient secretion of dengue virus non-structural protein 1

**DOI:** 10.1128/jvi.00962-25

**Published:** 2025-08-21

**Authors:** Stephen M. Johnson, Siena M. Centofanti, Gustavo Bracho, Michael R. Beard, Jillian M. Carr, Nicholas S. Eyre

**Affiliations:** 1 College of Medicine and Public Health (CMPH), Flinders University 1065, Bedford Park, South Australia, Australia; 2 Cell Screen SA, Flinders Centre for Innovation in Cancer, Flinders University 375963 , Bedford Park, South Australia, Australia; 3 Research Centre for Infectious Diseases, Department of Molecular and Biomedical Sciences, The University of Adelaide 1066, , Adelaide, South Australia, Australia; Wake Forest University School of Medicine, Winston-Salem, North Carolina, USA

**Keywords:** orthoflavivirus, flavivirus, dengue virus, NS1, NS1 secretion

## Abstract

Secreted non-structural protein 1 (sNS1) is an important orthoflavivirus pathogenic factor that can induce vascular leakage, a key symptom of severe dengue disease. Given the role of sNS1 in dengue pathogenesis, defining the molecular mechanisms of NS1 secretion may contribute to the development of NS1-targeting antiviral therapies. To this end, we performed a customized membrane-trafficking siRNA screen to identify human host factors involved in NS1 secretion. Our screen identified COPA, COPB2, and COPG1 as the top-ranking hits. These proteins are three of the seven subunits of the coatomer protein complex I (COPI) that coat transport vesicles that operate within the early secretory pathway, implicating COPI machinery as being involved in NS1 secretion. Validation studies employing host gene knockdown in dengue virus (DENV)-infected cells confirmed that COPI components are required for efficient NS1 secretion but are dispensable for infectious virus egress. Similar reductions in NS1 secretion were observed when COPI components were depleted in cells infected with West Nile virus Kunjin subtype (WNV/KUNV), indicating that the molecular mechanisms exploited to achieve NS1 secretion may be a conserved feature within the Orthoflavivirus genus. Heterologous expression of wild-type and pathogenic COPI variants in DENV NS1-NS5 polyprotein-expressing cells resulted in altered NS1 secretion profiles, suggesting that allelic variants and altered expression levels of COPI components may indirectly influence the severity of dengue disease. The identification of COPI components as important determinants of NS1 secretion efficiency may aid in the identification of novel targets for anti-orthoflaviviral therapies.

**IMPORTANCE:**

Over half of the world’s population is at risk of infection with mosquito-borne pathogenic orthoflaviviruses such as DENV. Although the secreted form of the viral NS1 protein has been identified as a major determinant of the pathogenic effects of DENV and related orthoflaviviruses, the exact mechanisms involved in NS1 secretion are poorly understood. Here, we interrogated host factors involved in the secretion of NS1 from infected cells using a customized membrane-trafficking siRNA screen. This revealed three components of the COPI complex that regulate vesicular transport in the early secretory pathway as important factors in NS1 secretion. The involvement of COPI components in NS1 secretion was further validated using wild-type DENV and WNV/KUNV infection, overexpression approaches, and chemical inhibition studies. Together, this study demonstrates the importance of COPI machinery in NS1 secretion and suggests that exploitation of this machinery in NS1 secretion may represent a future target of antiviral drug development.

## INTRODUCTION

Dengue virus (DENV) is the most prevalent arthropod-borne human viral pathogen, with half of the world’s population living in at-risk areas (1). It has been estimated that approximately 390 million DENV infections occur annually, with approximately 96 million infections resulting in disease (2). Symptoms range from mild febrile illness to life-threatening complications including dengue fever (DF), dengue hemorrhagic fever (DHF), and dengue shock syndrome (DSS). No dengue-specific antivirals are currently available.

DENV is an enveloped positive-sense single-stranded RNA virus with an approximately 11 kb genome that encodes three structural proteins (capsid, precursor-membrane, and envelope) and seven non-structural proteins (NS1, NS2A, NS2B, NS3, NS4A, NS4B, and NS5). Among these proteins, NS1 has attracted significant attention given its critical roles in viral RNA replication, infectious virus production, and disease pathogenesis (3–5). Translated in the endoplasmic reticulum (ER), the hydrophilic NS1 monomer has two N-linked glycosylation sites, N130 and N207, and exists briefly as a soluble monomer. NS1 monomers rapidly dimerize, forming a membrane-associated NS1 dimer, the predominant intracellular form (6, 7). Intracellular NS1 (iNS1) co-localizes with dsRNA at both the ER-lumenal surface and interior of the virus-induced replication organelles, where iNS1 plays an essential role in viral RNA replication (3, 8, 9). Recent evidence has demonstrated a role of iNS1 in virus particle assembly (10). NS1 dimers can also trimerize to form an open barrel-shaped NS1 hexamer that is stabilized by a lipid-rich central cavity and is efficiently secreted from the cell (11–13). Recent structural and biochemical studies, however, have revisited whether the extracellular sNS1 is hexameric, with studies reporting that sNS1 exhibits multiple oligomeric states, including tetramers and hexamers (14, 15), and as dimers in complex with host serum factors (16, 17). It is this secreted form of NS1 that has garnered much attention recently for its role as a virulence factor being implicated in dengue disease pathogenesis (5, 18, 19). Plasma sNS1 levels, which correlate with viremia in symptomatic individuals, are significantly higher in patients who develop DHF than DF (20). Antibodies elicited against NS1 have been shown to contribute both protective and pathogenic consequences (21), and sNS1 acts in immune evasion by interfering with complement pathways (22, 23). Importantly, sNS1 can induce the release of vasoactive cytokines from immune cells and can directly induce endothelial cell glycocalyx layer disruption and vascular leakage (24, 25), a key symptom of severe dengue. Given the pathological effects of sNS1, much research has been conducted on the synthesis, structure, and key functional residues of this multifunctional protein (10, 26–28). However, major gaps exist in our understanding of the mechanisms that drive NS1 secretion.

To identify human host cell factors that are involved in NS1 secretion, we performed a customized membrane-trafficking siRNA screen. Our screen identified components of the coatomer protein complex I (COPI), a cage-like protein complex that coats transport vesicles that are best known for the bidirectional trafficking of proteins and lipids within the early secretory pathway (29–32). Validation studies confirmed that COPI is required for efficient NS1 secretion, and that the exploitation of these components to achieve NS1 secretion may be a conserved feature within the Orthoflavivirus genus. Furthermore, we show that allelic variants of COPI may influence NS1 secretion profiles. This study expands our understanding of the molecular mechanisms of NS1 secretion and may aid in the identification of novel targets for anti-orthoflaviviral therapies.

## RESULTS

### A customized membrane-trafficking siRNA screen implicates COPI components as important determinants of DENV NS1 secretion

**Fig 1:**
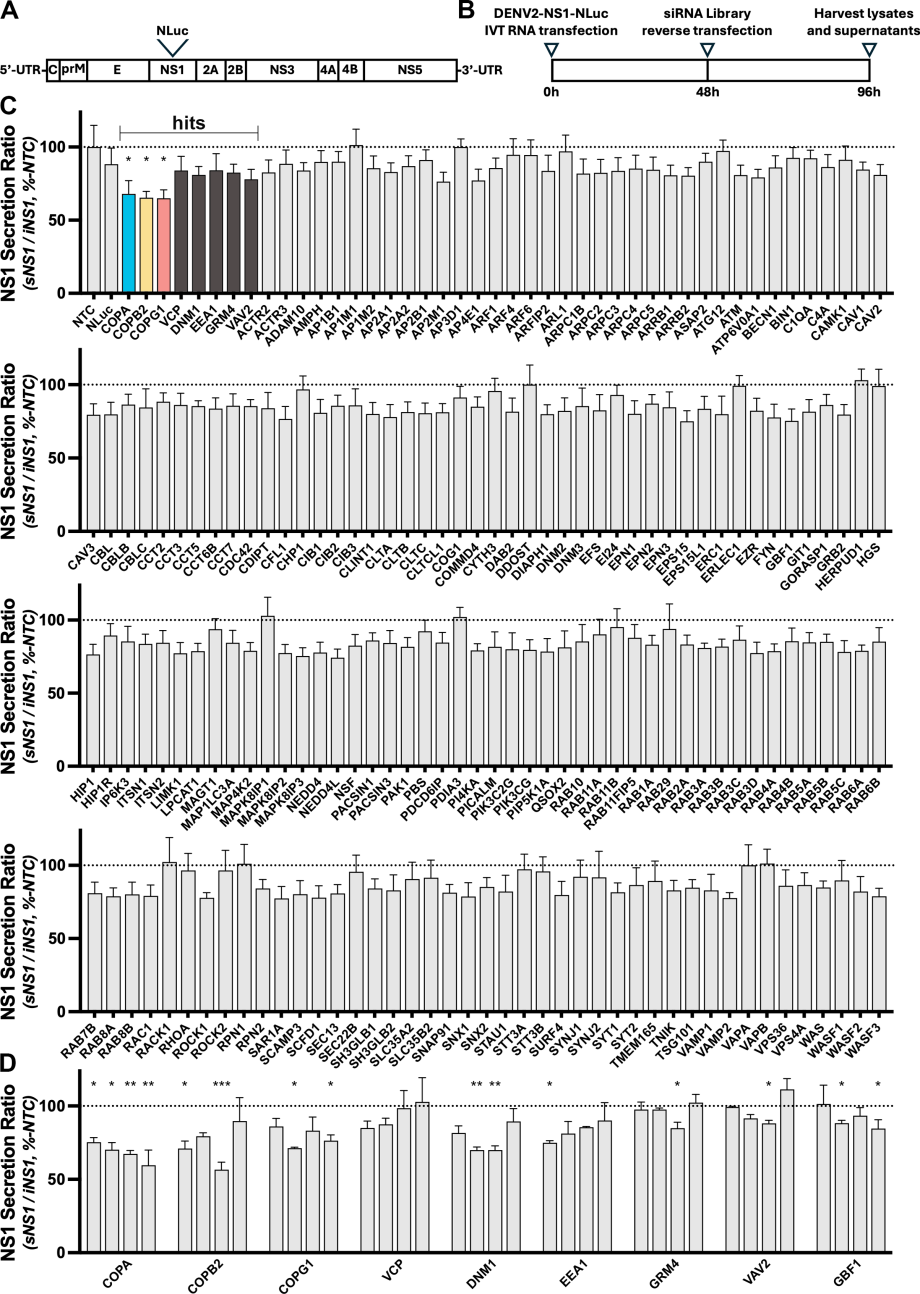
A customized membrane-trafficking siRNA screen implicates COPI components as important determinants of NS1 secretion. (**A**) Schematic overview of the infectious DENV2 construct bearing a NanoLuc luciferase (NLuc) tag within NS1. (**B**) Schematic overview of the high-throughput siRNA screen strategy. (C and D) Secretion ratio of NS1-NLuc (supernatant NS1-NLuc / lysate NS1-NLuc) as a % of average values associated with a non-targeting control (NTC) siRNA for the customized membrane-trafficking screen involving siRNA pools for each gene of interest (**C**) and deconvolution screen involving testing of the four individual siRNA duplexes that comprised the siRNA pools for hits identified in the primary screen, compared to the average values of those of the NTC control siRNA pool (**D**). For the membrane-trafficking siRNA screen (**C**), of each experimental siRNA pool, data are means + S.D. from nine measurements from three independent experiments. For the identified hits, the significance of differences between siNTC controls and the indicated siRNA pools was determined using unpaired (two-tailed) *t*-tests, *P <0.05. Hit scores, as defined in the supplemental material, were as follows: COPA (score = 13), COPB2 (score = 13), COPG1 (score = 12), VCP (score = 7), DNM1 (score = 5), EEA1 (score = 5), GRM4 (score = 5), and VAV2 (score = 5). For the deconvolution screen (**D**), each experimental siRNA duplex that constituted the respective pool, data are means + S.D. from 12 (siNTC control) or 3 (individual siRNA duplexes) replicates from one experiment. The significance of differences between siNTC controls and the indicated siRNA duplexes was determined using unpaired (two-tailed) *t*-tests, *P <0.05, ** *P* <0.01, ***P <0.001. Hit criteria for these siRNA screens, which are also involved in the determination and analysis of siRNA effects on cell viability, are described in the supplemental material.

sNS1 is an important DENV pathogenic factor. Defining the molecular mechanisms of NS1 secretion may contribute to the development of NS1-targeting antiviral therapies. To identify human host cell factors involved in DENV NS1 secretion, we utilized an infectious DENV2-NS1-NLuc reporter virus construct that bears the small and sensitive NanoLuc luciferase (NLuc) reporter embedded within NS1 (8) (Fig. 1A). This reporter virus allows for the simple and reliable quantification of intracellular and secreted NS1-associated NLuc activity in infected cell cultures and is amenable to high-throughput screening.

The DENV2-NS1-NLuc reporter virus was used to screen a membrane-trafficking siRNA library (Dharmacon; 140 genes) that was customized and curated to include additional siRNA pools for 37 host genes, which have been identified in previous studies as important host-dependency factors that may be manipulated by NS1 (33, 34). Each siRNA pool targeting the ~180 host genes consists of four individual siRNA duplexes that recognize different sequences within each target transcript. Figure 1B provides a schematic overview of the siRNA screen strategy. Briefly, Firefly luciferase (FLuc)-expressing Huh-7.5 cells were transfected with *in vitro* transcribed DENV2-NS1-NLuc RNA and cultured for 48 hours to establish infection. Cells were then trypsinized and reverse transfected with the individual siRNA library pools in 96-well plates and returned to culture for a further 48 hours. Cell culture lysates and supernatants were harvested and assayed for intracellular and secreted NS1-associated NLuc activity, respectively, and normalized to intracellular Firefly luciferase activity, which was used as an indicator of cell viability. The data analysis methods and hit selection criteria are detailed in the supplemental material.

Only one experimental siRNA pool, RHOA, reproducibly reduced cell viability-associated FLuc luciferase levels to 1 standard deviation below the mean of the NTC. As such, any impact of RHOA on NS1 secretion was not considered further in this study. Compared to non-targeting siRNA controls, we identified eight siRNA pools that inhibited NS1-NLuc secretion without significantly affecting cell viability. Figure 1C shows the extracellular NS1-NLuc-to-intracellular NS1-NLuc ratios (sNS1-NLuc / iNS1-NLuc), a measure of NS1 secretion efficiency, from the three independent siRNA screen repeats. Interestingly, the top three hits, COPA, COPB2, and COPG1, are three of the seven subunits of the coatomer protein complex I (COPI), a heptameric protein matrix that coats transport vesicles that operate within the early secretory pathway.

To validate the siRNA screen hits, a deconvolution screen was performed. Here, each of the four constituent siRNA duplexes from each pool was screened individually. While not identified as a hit in the screen, GBF1 was also included in this deconvolution screen due to its role as a master regulator of COPI vesicle formation (35). GBF1 is a guanine nucleotide exchange factor (GEF) that catalyzes the GDP to GTP exchange on ADP-ribosylation factors (ARFs). GTP-activated ARFs then recruit preassembled heptameric COPI complexes to donor membranes to form COPI-coated vesicles (29). For several of the nine gene targets, at least two siRNAs decreased NS1 secretion as inferred from the NS1-NLuc secretion ratio (Fig. 1D). Of the COPI components COPA, COPB2, and COPG1, at least two of the individual siRNAs reduced the NS1-NLuc secretion ratio, providing further support that COPI machinery is involved in NS1 secretion. Taken together, several genes that encode components of the multi-subunit COPI complex were identified as critical factors involved in DENV NS1 secretion.

### NS1 secretion is reduced in COPI-silenced Huh-7.5 cells

Following the identification of components of the COPI complex as putative determinants of efficient NS1 secretion, we next sought to confirm the effects of siRNA-mediated COPI gene knockdown and associated effects on NS1 secretion using wild-type infectious DENV2 and the related orthoflavivirus, Australian endemic West Nile virus Kunjin subtype (WNV/KUNV).

**Fig 2:**
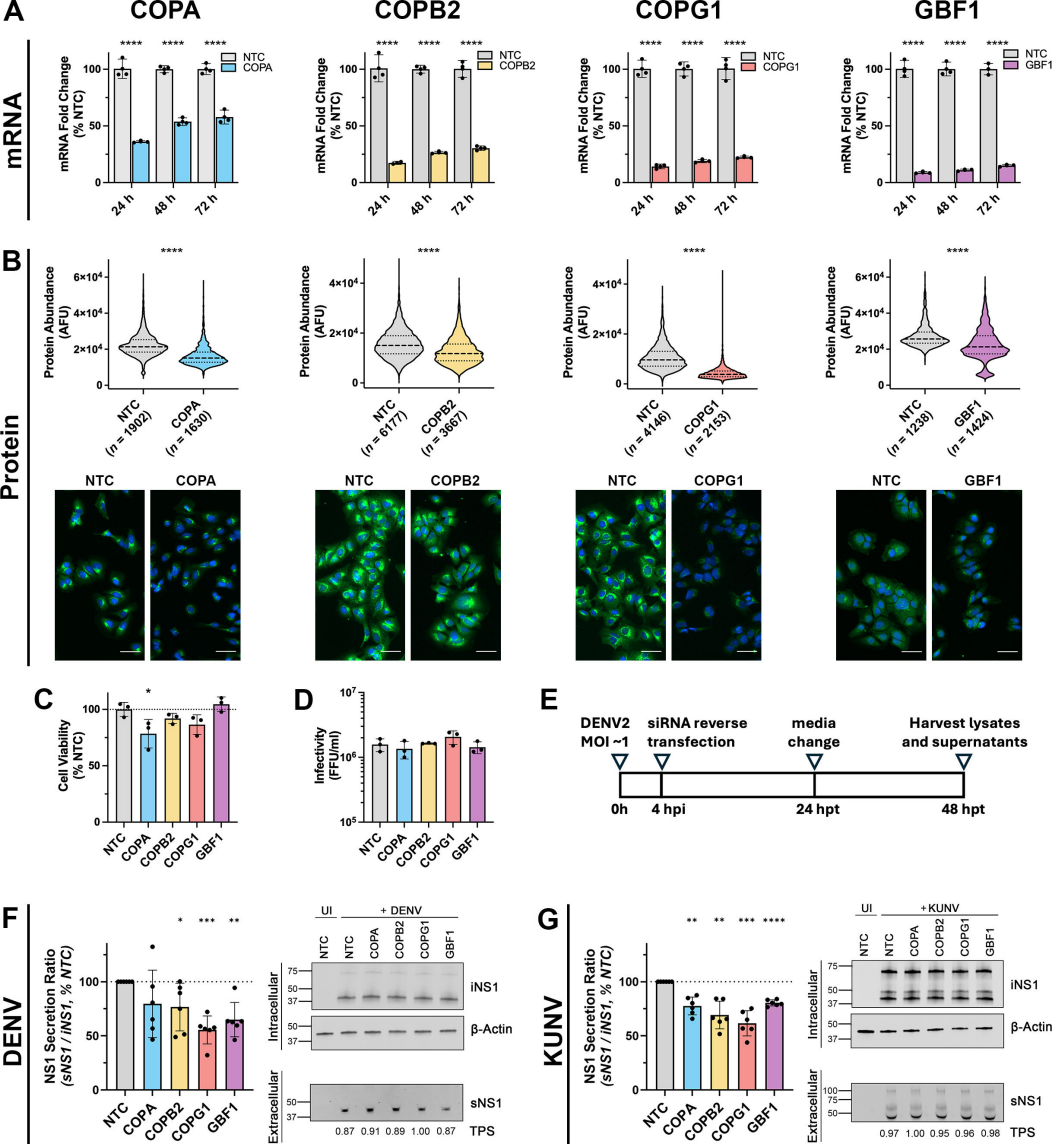
DENV and WNV/KUNV NS1 secretion is reduced in COPI-silenced Huh-7.5 cells. (**A**) qRT-PCR analysis of COPI component mRNA levels in Huh-7.5 cells at indicated time points following siRNA treatment. Data are normalized to those of the RPLP0 housekeeping gene and expressed as a % of those of the non-targeting control (NTC) siRNA. Data are means +S.D., *n* = 3, one-way ANOVA. (**B**) Immunofluorescence microscopy-based quantitative analysis of COPI component protein abundance in Huh-7.5 cells following siRNA treatment. Huh-7.5 cells were reverse transfected with COPI siRNA pools or the NTC siRNA pool as indicated. At 48 h.p.t., cells were fixed and processed for indirect immunofluorescent labeling using anti-COPI antibodies (green), and nuclei were counterstained with DAPI (blue). Scale bars, 50 µm. Fluorescence intensity was measured for each cell to determine COPI protein abundance at the single-cell level. Violin plots (with light smoothing) display median values (dashed lines) and quartile values (dotted lines) for each data set. Mean fluorescence intensity as a percentage of NTC is displayed on the x-axis. Cell numbers (N) COPA: NTC = 1902, COPA = 1630; COPB2: NTC = 6177, COPB2 = 3667; COPG1: NTC = 4146, COPG1 = 2153; GBF1: NTC = 1238, GBF1 = 1424. The statistical significance of differences between groups was determined using Welch’s t-test. (**C**) DENV-infected Huh-7.5 cell viability and infectious virus production is largely unaffected by COPI silencing. Huh-7.5 cells were infected with DENV2 (MOI ~1) for 4 h and reverse transfected with siRNAs targeting the indicated COPI component or NTC. At 48 hours post-siRNA treatment, cell viability was measured using a CellTiter-Glo 2.0 cell viability assay (**C**), and virus-containing cell culture supernatants were recovered and processed to assess infectivity by focus forming assays (**D**). Data are means +S.D., *n* = 3 biological triplicates, one-way ANOVA. (**E**) Schematic overview of the experimental approach to assess the impact of COPI silencing on wild-type DENV2 or WNV/KUNV NS1 secretion. Huh-7.5 cells were infected with DENV2 or WNV/KUNV (MOI ~1), trypsinized at 4 h.p.i., and reverse transfected with siRNAs targeting the indicated COPI components or NTC. At 24 hours post-siRNA treatment, cells were washed, and media was replaced. At 48 hours post-siRNA treatment, cell culture supernatants and lysates were recovered to measure extracellular and intracellular NS1 levels, respectively, by quantitative Western blot analysis. (F–G) Quantification of NS1 abundance in cell culture supernatants (normalized to TPS values) and lysates (normalized to β-actin values) by Western blot analysis, displayed as the secretion ratio of NS1 (sNS1/iNS1) as a % of NTC. Western blot images depict detection of intracellular NS1 (iNS1) protein and the corresponding loading control protein β-actin (upper panels) and secreted NS1 (sNS1) and corresponding loading control Total Protein Stain values (TPS; values under the sNS1 blot are expressed as a fold-change of that of the siNTC control) for supernatant samples (lower panels). Images from representative Western blot experiments are shown for DENV (**F**) and WNV/KUNV (**G**). Data are means ±S.D., *n* = 3 from two independent experiments, one-sample *t*-test. *P <0.05, **P <0.005, ***P ≤0.0005, ****P <0.0001.

After validating the efficacy of our siRNAs in the knockdown of the expression of their target mRNA in Huh-7.5 cells (Fig. 2A), we confirmed that COPI siRNA treatment successfully reduced target protein abundance by quantitative indirect immunofluorescence microscopy, using fluorescence intensity as a readout of protein abundance (Fig. 2B). Importantly, DENV-infected Huh-7.5 cell viability was largely unaffected by COPI siRNA treatment (Fig. 2C), as determined using an ATP-based cell viability assay. Infectious virus production was unaltered by COPI silencing (Fig. 2D), suggesting that COPI siRNA treatment does not impair DENV RNA replication, virion assembly, or virus egress when COPI gene knockdown is applied at 4 hours post-DENV infection. To assess the impact of COPI silencing on NS1 secretion, Huh-7.5 cells were infected with DENV for 4 hours and trypsinized and reverse transfected with siRNA pools targeting COPI components. At 24 hours post-infection (h.p.i.), cell culture media was replaced, and cell culture lysates and supernatants were harvested at 48 h.p.i. to assess intracellular and secreted NS1 abundance, respectively, by quantitative Western blot analysis (Fig. 2E). Under each of the COPI and GBF1 siRNA treatments, DENV NS1 secretion was reduced as inferred from the NS1 secretion ratios (sNS1/iNS1) relative to cells transfected with the non-targeting siRNA control (Fig. 2F), reflecting the results of the original siRNA screen. Similarly, a reduction in NS1 secretion was observed when these COPI components and GBF1 were depleted in Huh-7.5 cells infected with WNV/KUNV (Fig. 2G).

**Fig 3:**
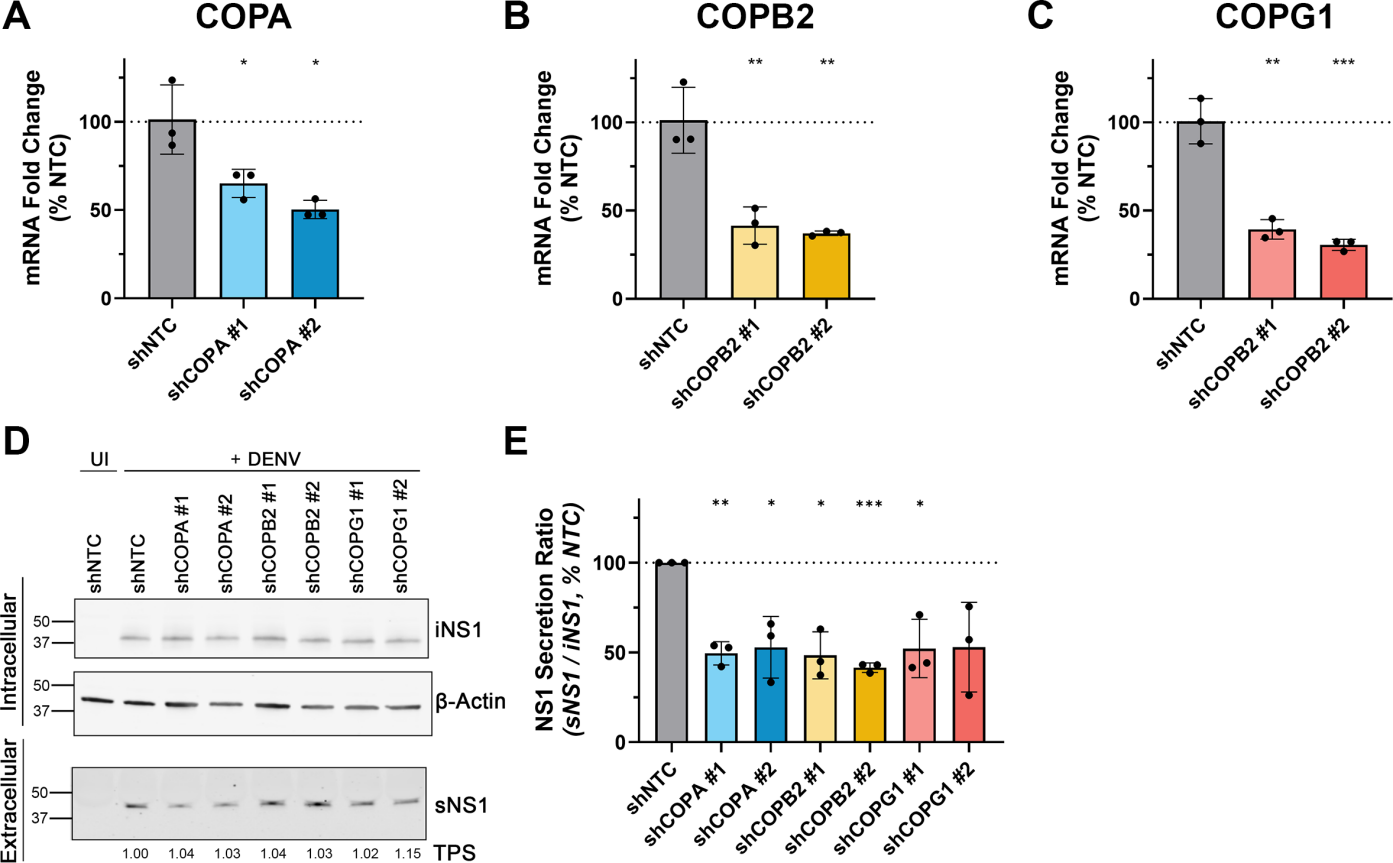
Knockdown of COPA, COPB2, and COPG1 via shRNA expression reduces DENV-2 NS1 secretion efficiency. (**A–C**) Stable Huh-7.5 cell lines that displayed doxycycline-dependent expression of a non-targeting control (NTC) shRNA or shRNAs that were specific for COPA, COPB2, or COPG1 (two different shRNA sequences per gene; #1 and #2) were cultured in the presence of doxycycline (1 µg/mL) for 72 h before extraction of total cellular RNA and qRT-PCR analysis of (**A**) *COPA*, (**B**) *COPB2,* and (**C**) *COPG1* mRNA levels. For each COPI target gene, mRNA levels were normalized to those of the *RPLPO* housekeeping gene and expressed as a percentage of average shNTC control values. Data are means ±S.D. (*n* = 3), unpaired (two-tailed) *t*-tests, *P <0.05, **P <0.01, ***P <0.001. (**D-E**) Quantitative Western blotting analysis of DENV-2 NS1 protein secretion. The indicated stable shRNA-expressing Huh-7.5 cell lines were cultured in the presence of doxycycline (1 µg/mL) overnight and then infected with DENV2 (MOI: ~0.1). At 24 hours post-infection, cells were washed and returned to culture (in the presence of doxycycline) for a further 24 hours before cell culture supernatants and lysates were recovered to measure extracellular and intracellular NS1 levels, respectively, by quantitative Western blot analysis. (**D**) Western blot images for detection of intracellular NS1 (iNS1) protein and the corresponding loading control protein β-actin (upper panels) and secreted NS1 (sNS1) and corresponding loading control Total Protein Stain values (TPS; values under the sNS1 blot are expressed as a fold-change of that of the shNTC control) for supernatant samples (lower panels). Images from a representative Western blotting experiment are shown. (**E**) Quantification of NS1 abundance in cell culture supernatants (normalized to TPS values) and lysates (normalized to β-actin values), displayed as the secretion ratio of normalized NS1 (sNS1/iNS1), as a % of NTC. Data are means ±S.D., *n* = 3, one-sample *t*-test. *P <0.05, **P <0.01, ***P <0.001.

To further validate the involvement of COPA, COPB2, and COPG1 in efficient DENV NS1 secretion, lentiviral vectors were generated and employed to generate stable Huh-7.5 cell lines displaying doxycycline-inducible expression of microRNA-adapted shRNA that were designed to target these genes. Following the validation of target gene knockdown by qRT-PCR (Fig. 3A through C), DENV-2 infection and quantitative Western blot analysis were employed to investigate the impact of shRNA-mediated COPA, COPB2, and COPG1 knockdown on NS1 secretion efficiency. This analysis revealed significantly reduced NS1 secretion ratios relative to those of a cell line expressing a non-targeting control (NTC) shRNA (Fig. 3D and E). While both siRNA- and shRNA-mediated approaches for knockdown of target COPA, COPB2, and COPG1 gene expression resulted in significant inhibition of NS1 protein secretion efficiencies, the magnitude of each mRNA knockdown, as determined by qRT-PCR, and the corresponding effects on NS1 protein secretion efficiency were not always consistent between the different RNAi approaches. For example, while the quantitative Western blotting experiments indicated that siRNA-mediated knockdown of COPG1 expression had the greatest effects on NS1 protein secretion efficiency, similar experiments using stable shRNA cell lines indicated that the relative importance of COPG1 to NS1 secretion efficiency was similar to that of COPA and COPB2. These discrepancies may be attributable to experimental variability, differences in the experimental systems, and/or differences in the homogeneity, degree, and timing of protein knockdown for the various target genes and respective RNAi approaches. Taken together, these data indicate that COPI components and GBF1 are required for efficient DENV NS1 secretion but are dispensable for infectious virus secretion. Furthermore, the exploitation of COPI components and GBF1 to achieve NS1 secretion from human cells may be a conserved feature within the Orthoflavivirus genus.

### Heterologous expression of COPI variants suggests that certain variants and overexpression affect DENV NS1 secretion

**Fig 4:**
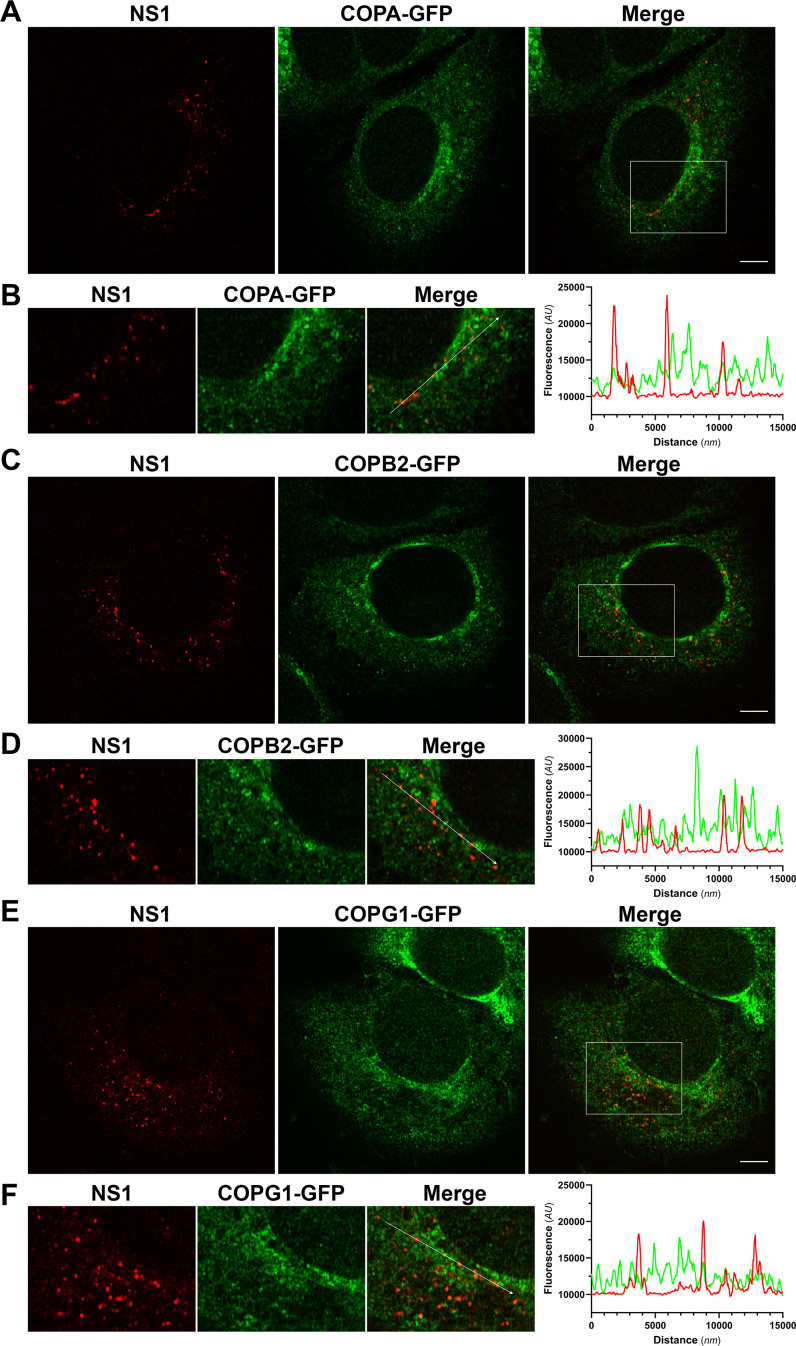
Confocal analysis of COPA, COPB2, and COPG1 localization reveals minimal and infrequent colocalization with NS1 protein in infected cells. Huh-7.5 cells stably expressing GFP-tagged COPA (**A and B**), COPB2 (**C and D**), or COPG1 (**E and F**) were infected with DENV2 (MOI ~1) for 24 h prior to fixation and indirect immunofluorescent labeling using anti-NS1 (red) and anti-GFP (green) antibodies. Samples were processed for Airyscan confocal imaging and appropriate image processing, as described in Materials and Methods. Representative images, single central z-sections, and merged images are shown, as indicated. Image panels in B, D, and F depict “zoom insets” taken from the indicated white box areas in Merge images in A, C, and E, respectively. Graphs in B, D, and F depict 15 µm line profiles for the lines indicated by white arrows in the corresponding “zoom inset” Merge images. Scale bars, 10 µm.

Since siRNA- and shRNA-mediated depletion of COPI components reduced NS1 secretion in DENV-infected human cells, we next explored potential interactions between DENV2 NS1 and COPI components by immunofluorescence and confocal microscopy analysis. Huh-7.5 cells stably expressing GFP-tagged COPA, COPB2, or COPG1 cDNA were infected with DENV2 (MOI ~0.1) for 24 hours prior to fixation, indirect immunofluorescent labeling using anti-NS1 and anti-GFP antibodies, and confocal microscopy. While GFP-tagged COPI components displayed intense juxtanuclear and vesicular cytoplasmic staining patterns, consistent with Golgi-like localization, these studies revealed infrequent colocalization between cytoplasmic NS1 foci and COPI vesicle marker staining (Fig. 4A, C, and E), as highlighted by rare and incidental overlap of NS1- and COPI-associated peaks in line profile analyses (Fig. 4B, D and F). These results indicate that the vast majority of intracellular NS1 does not colocalize with and is spatially distinct from COPI components, suggesting that potential interactions between secretion-destined NS1 and COPI may be infrequent and/or transient in nature. Alternatively, it is possible that COPI components play an indirect role in orthoflavivirus NS1 protein secretion.

**Fig 5:**
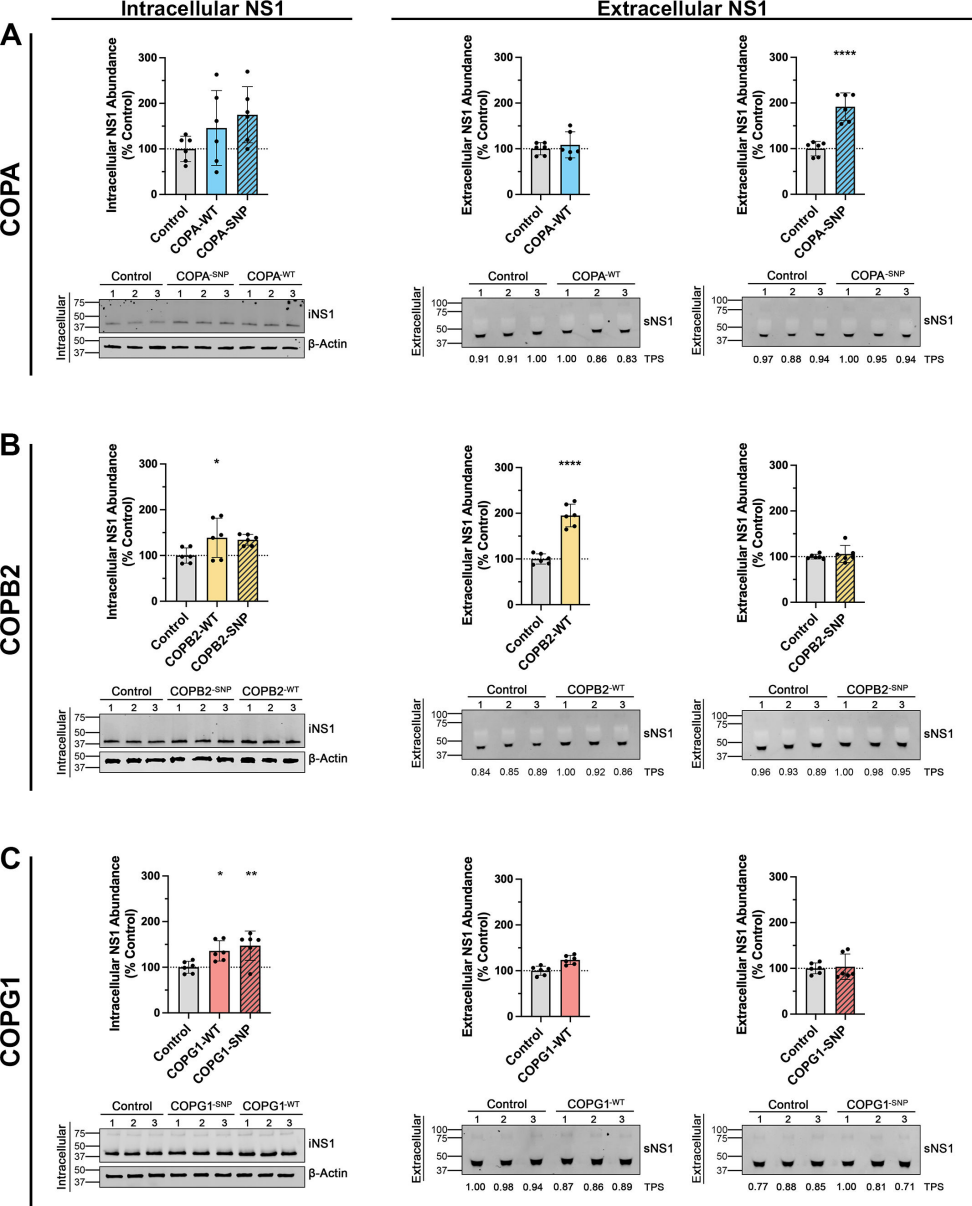
Heterologous expression of COPI variants indicates that certain variants and overexpression affect DENV2 NS1 secretion. Single-nucleotide polymorphisms (SNPs) were introduced into GFP-tagged wild-type COPA, COPB2, and COPG1 cDNA expression constructs. T7 RNA polymerase-expressing Huh-7.5 cells were co-transfected with COPI expression constructs and a T7 RNA polymerase-driven DENV2 NS1-NS5 polyprotein expression system. At 18 hours post-transfection, cell culture supernatants and lysates were recovered to measure extracellular and intracellular NS1 levels, respectively, by quantitative Western blot analysis. Western blot images depict detection of intracellular NS1 (iNS1) protein and the corresponding loading control protein β-actin (left panels) and secreted NS1 (sNS1) and corresponding loading control Total Protein Stain values (TPS; values under the sNS1 blots are expressed as a fold-change of that of the empty vector control) for supernatant samples (right panels). The numbers above the blots indicate experimental replicate samples. Data are means + SD, *n* = 3 from two independent experiments, one-way ANOVA, *P <0.05, **P <0.01, ***P <0.005, ****P <0.0001.

Next, we sought to perturb the COPI pathway using an RNA interference (RNAi)-independent approach. In line with previous studies (36, 37), our attempts to generate COPI component knockout cell lines using CRISPR/Cas9 failed to yield cells completely deficient in COPI component protein (data not shown), likely due to the established roles of these genes for optimal cell proliferation (38). However, medically relevant loss-of-function single-nucleotide polymorphisms (SNPs) have recently been identified for COPA^-E241K^ (39), COPB2^-R254C^ (40), and COPG1^-K652E^ (41). Accordingly, we investigated the impact of overexpression of wild-type and SNP variants of these genes on NS1 secretion efficiency. Importantly, COPA^-E241K^ is a dominant negative variant (39); therefore, heterologous cDNA expression should interfere with the proper functioning of the endogenously expressed wild-type protein. To date, no dominant negative SNP mutations have been identified for COPB2 or COPG1. Indeed, COPB2^-R254C^ and COPG1^-K652E^ are homozygous recessive mutations. Nevertheless, these variants are incorporated into COPI complexes, resulting in impaired COPI function (41). To this end, GFP-tagged wild-type COPA, COPB2, and COPG1 cDNA constructs were generated, and modified variants were generated by site-directed mutagenesis to incorporate these SNPs. To examine the effects of COPI^-WT^ or COPI^-SNP^ overexpression on NS1 secretion, independent of viral RNA replication and/or spread of infection, we employed the T7 RNA polymerase-driven pIRO-D expression system in which heterologously expressed T7 RNA polymerase drives the expression of the DENV2 NS1-NS5 polyprotein, and induces the formation of replication organelles that are morphologically indistinguishable from those of wild-type DENV infection (42). COPI^-WT^, COPI^-SNP^, or GFP-only control plasmids was co-transfected with pIRO-D into T7 RNA polymerase-expressing Huh-7.5 cells. At 18 hours post-transfection, cell culture lysates and supernatants were collected to assess the impact of COPI^-WT^ and COPI^-SNP^ cDNA over-expression on intracellular and secreted NS1 abundance by quantitative Western blot analysis. Despite substantial variability of iNS1 levels when either COPA^-WT^ or COPA^-E241K^ cDNA was expressed, sNS1 levels were relatively consistent within treatment groups (Fig. 5A). Interestingly, while COPA^-WT^ overexpression had no effect on sNS1 levels, expression of the COPA^-E241K^ construct increased sNS1 levels approximately twofold. Modest increases in iNS1 abundance were observed when either COPB2^-WT^ or COPB2^-R254C^ constructs were over-expressed; however, an approximately twofold increase in sNS1 abundance was observed in COPB2^-WT^-transfected cells (Fig. 5B). Similarly, modest increases in iNS1 were observed in cells transfected with either COPG1^-WT^ or COPG1^-K652E^ constructs; however, no effect was observed for levels of sNS1 (Fig. 5C). Collectively, the altered NS1 secretion profiles observed here for COPA^-E241K^ and COPB2^-WT^ suggest that allelic variants and/or altered expression levels of COPI components may enhance DENV NS1 secretion.

**Fig 6:**
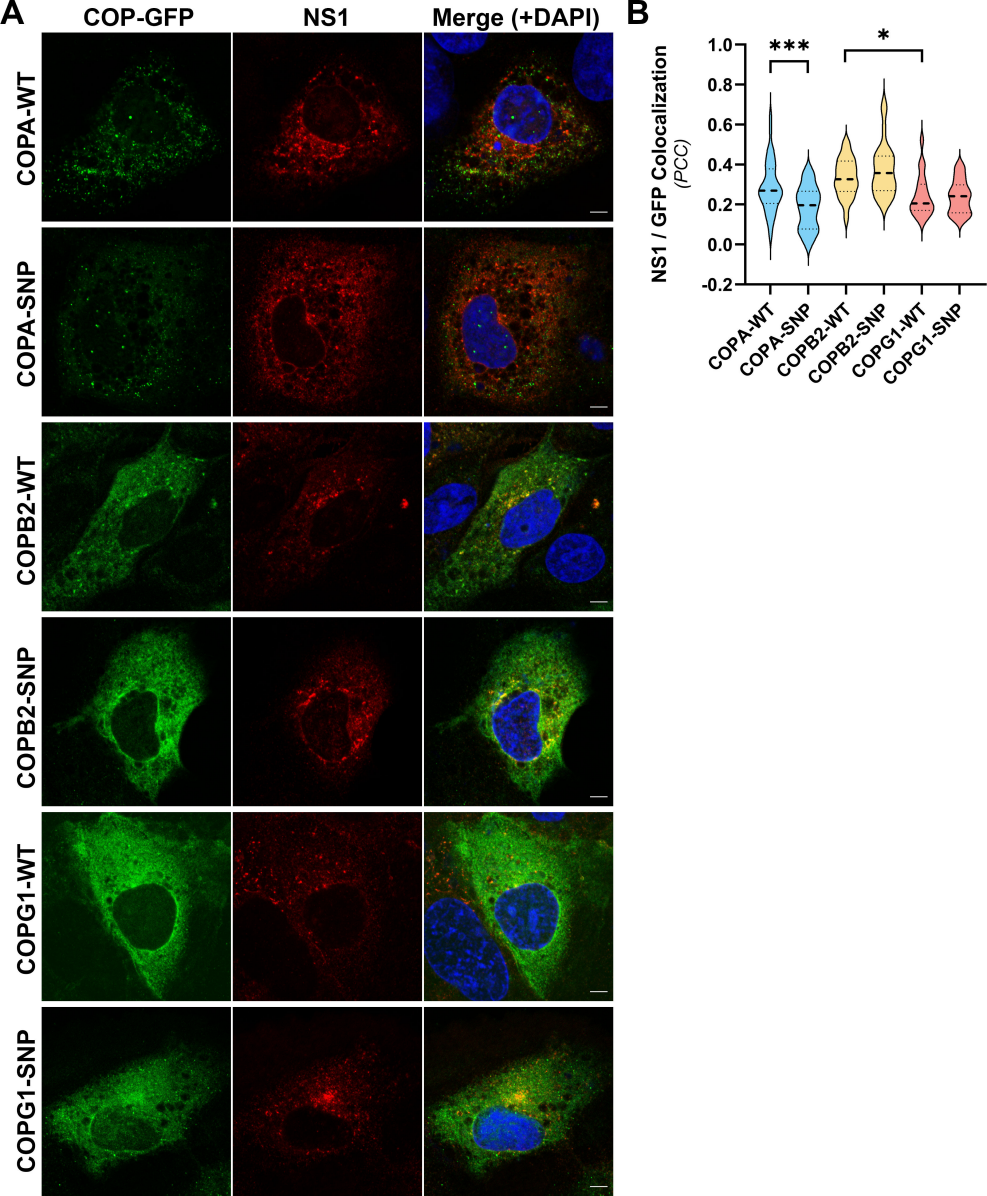
The subcellular localization of heterologously expressed NS1 relative to that of wild-type and SNP variants of COPA, COP2, and COPG1. (**A**) T7 RNA polymerase-expressing Huh-7.5 cells were co-transfected with the indicated GFP-tagged COPI expression constructs and a T7 RNA polymerase-driven DENV2 NS1-NS5 polyprotein expression system. At 24 hours post-transfection, cells were fixed and processed for indirect immunofluorescent labeling using mouse anti-NS1 and rabbit anti-GFP primary antibodies, followed by AlexaFluor 555-conjugated anti-mouse IgG (red) and AlexaFluor 488-conjugated anti-rabbit IgG (green). Samples were counterstained with DAPI and analyzed by confocal fluorescence microscopy. Yellow in the merged images indicates co-localization. Scale bars are 5 µM. (**B**) Violin plots of Pearson’s co-localization coefficients for individual cells stained for both NS1 and the indicated COP-GFP proteins. Dashed lines indicate median values, while dotted lines indicate quartiles (*n* ≥ 20 cells/group). Statistical significance was determined using a two-way ANOVA test and Sidak’s multiple comparisons (noting that statistically significant differences between the different COP-SNP proteins are not indicated), *P <0.05, ***P <0.001.

To complement these studies and further explore the colocalization of NS1 with these COPI components and the impact of SNP variants on this colocalization, quantitative confocal fluorescence microscopy was employed. Specifically, COPI^-WT^ and COPI^-SNP^ expression plasmids were co-transfected with pIRO-D into T7 RNA polymerase-expressing Huh-7.5 cells, and cells were fixed at 24 hours post-transfection and processed for indirect immunofluorescent labeling of NS1 and GFP. Consistent with earlier experiments in the context of DENV-2 infection (Fig. 3), confocal analysis revealed very infrequent and modest levels of colocalization between NS1 and COPI components (Fig. 6A and B). However, quantitation of colocalization revealed that COPA^-E241K^ colocalization with NS1 was significantly lower than that of wild-type COPA, while SNP variants of COPB2 and COPG1 were not associated with significantly altered levels of NS1 colocalization as compared to their wild-type counterparts (Fig. 6B). It was also apparent that both wild-type and SNP variants of COPB2 and COPG1 displayed more reticular subcellular staining patterns than that of COPA and COPA^-E241K^, which displayed more vesicular staining patterns (Fig. 6A). While similar, colocalization of NS1 with COPB2 was significantly greater than that of NS1 with COPG1 (Fig. 6B). While we also observed that COPB2^-R254C^ displayed significantly greater levels of colocalization with NS1 than that of COPA^-E241K^ and COPG1^-K652E^ (not indicated in Fig. 6B), the meaningfulness of such differences is difficult to interpret, given the differing nature of these mutations.

### NS1 secretion is reduced in Golgicide A-treated Huh-7.5 cells

**Fig 7:**
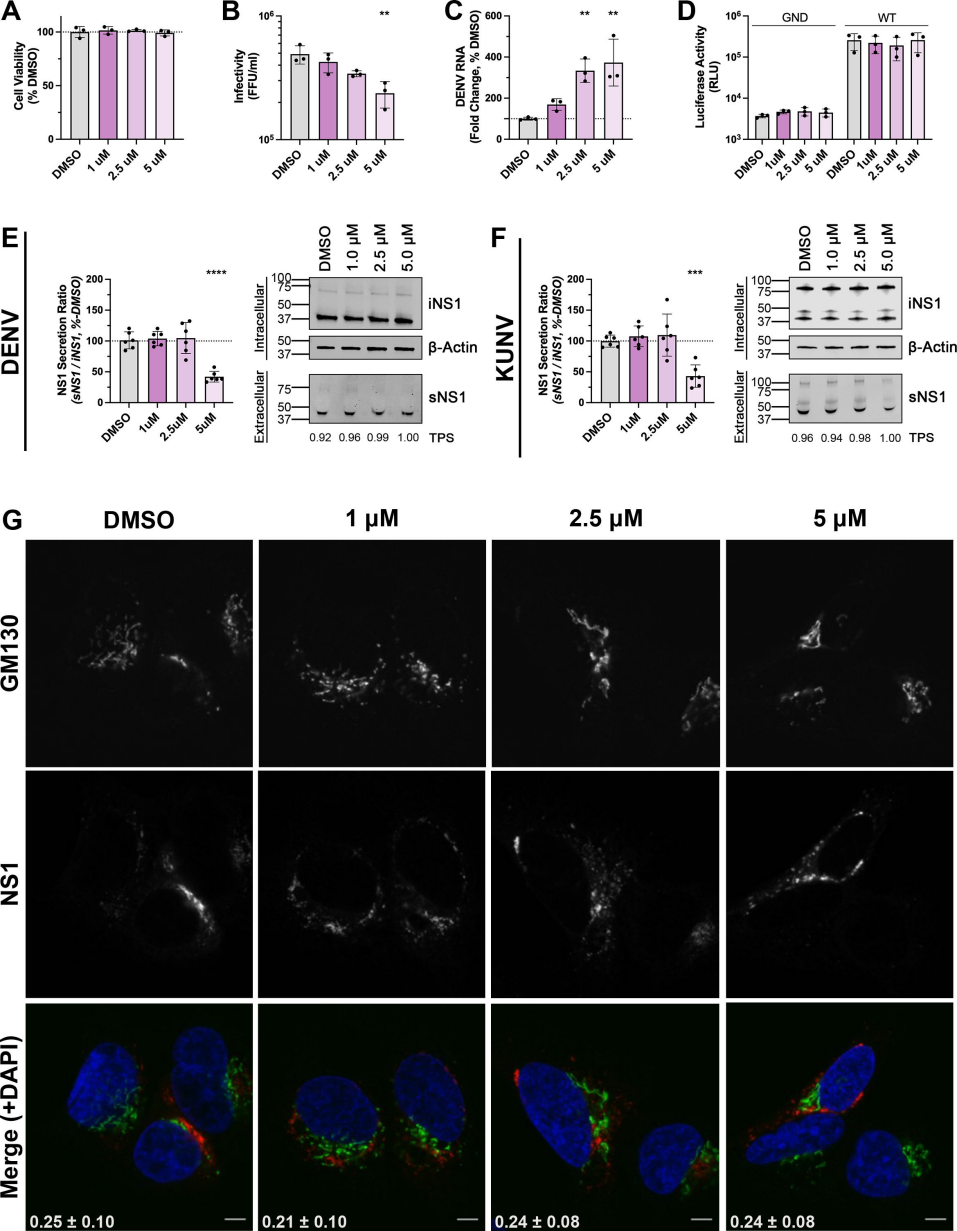
DENV2 and WNV/KUNV NS1 secretion is reduced in Golgicide A-treated Huh-7.5 cells. (**A–C**) Impact of Golgicide A (GCA) on DENV biology. Huh-7.5 cells were infected with DENV2 (MOI ~1). At 24 hours post-infection, cells were washed and cultured for a further 18 h in media supplemented with increasing concentrations of GCA or DMSO control. At 18 hours post-GCA treatment, cell viability was measured using CellTiter-Glo 2.0 viability assay (**A**), virus-containing cell culture supernatants were recovered and processed to assess infectivity by focus forming assay (**B**), and total cellular RNA was collected for qRT-PCR analysis of DENV2 viral RNA levels. For qRT-PCR analysis, data are normalized to the RPLP0 housekeeping gene and expressed as a % of non-targeting control (NTC) siRNA-treated mean values (**C**). Data are means + SD, *n* = 3 biological triplicates. (**D**) Golgicide A does not impact DENV RNA replication. Huh-7.5 cells were transfected with *in vitro* transcribed (IVT) RNA for a DENV2 subgenomic reporter replicon sg-DVs-R2A (WT, or replication-deficient GND control). At 4 hours post-transfection, cells were lysed (4 h timepoint) or cultured in GCA at the indicated concentration or DMSO carrier control. At 18 h post-GCA treatment, cell lysates were prepared and luciferase activities were determined as a surrogate marker for viral RNA replication. Data are means + SD, *n* = 3 biological triplicates. (E–F) Quantification of NS1 abundance in cell culture supernatants (normalized to TPS values) and lysates (normalized to β-actin values), displayed as the secretion ratio of NS1 (sNS1/iNS1) as a % of DMSO. Data are means + SD, *n* = 3 from two independent experiments, one-way ANOVA, **P <0.005, *** *P* <0.001, ****P <0.0001. Western blot images depict detection of intracellular NS1 (iNS1) protein and the corresponding loading control protein β-actin (upper panels) and secreted NS1 (sNS1) and corresponding loading control Total Protein Stain values (TPS; values under the sNS1 blot are expressed as a fold-change of that of the DMSO control) for supernatant samples (lower panels). Images from representative Western blot experiments are shown for DENV (**E**) and WNV/KUNV (**F**). (**G**) Localization of NS1 with respect to the Golgi marker GM130. Huh-7.5 cells were cultured as shown in E. At 18 hours post-GCA treatment, cells were fixed and stained for indirect immunofluorescent labeling using mouse anti-NS1 and rabbit anti-GM130 primary antibodies, followed by AlexaFluor 555-conjugated anti-mouse IgG (red) and AlexaFluor 488-conjugated anti-rabbit IgG (green). Samples were counterstained with DAPI and analyzed by confocal fluorescence microscopy. Yellow in the merged images indicates co-localization. Pearson’s co-localization coefficients are shown in white in the merged images (means + SD, n = >30 cells). Scale bars are 10 µM.

Golgicide A (GCA) is a potent and specific inhibitor of GBF1 catalytic activity that acts by binding to the GBF1-Arf-GDP protein-protein interface, preventing the Arf-GDP/GTP exchange (43). This results in COPI dissociation from Golgi membranes, prevention of COPI vesicle formation, disassembly of the Golgi, and swelling of the ER (43). GBF1 has been demonstrated to play a variety of roles in many RNA virus lifecycles (44), and much of this information has been elucidated by the use of GCA or the related multi-ArfGEF inhibitor brefeldin A (BFA) (45–49). To further investigate the effect of GCA on various aspects of DENV biology, Huh-7.5 cells were infected with DENV for 24 hours. At 24 h.p.i., cell culture media was replaced with GCA-supplemented media (0–5 µM) and returned to culture for a further 18 hours prior to analysis. First, we tested the effect of GCA treatment on DENV-infected Huh-7.5 cell viability using an ATP-based cell viability assay. No significant effects on cell viability were observed at GCA concentrations ≤ 5 µM (Fig. 7A). Increasing concentrations of GCA from 1 to 5 µM did, however, reveal a dose-dependent reduction of infectious virus production (Fig. 7B), accompanied by increases in intracellular viral RNA abundance (Fig. 7C). The use of a *Renilla* luciferase-encoding subgenomic replicon confirmed that this increase in intracellular viral RNA abundance was not the result of changes to viral RNA replication (Fig. 7D). These results suggest that GCA-mediated GBF1 inhibition does not influence DENV genome replication but, instead, impedes infectious DENV particle release when GCA is applied to cells after 24 hours of DENV infection. To assess the impact of GCA on NS1 secretion, cell culture lysates and supernatants were recovered from DENV-infected, GCA-treated Huh-7.5 cells. A decrease in NS1 secretion, as inferred from the NS1 secretion ratio (sNS1/iNS1), was observed in cells treated with 5 µM GCA, indicating that GCA reduces NS1 secretion from DENV-infected Huh-7.5 cells (Fig. 7E). Similar reductions in NS1 secretion were observed for WNV/KUNV-infected Huh-7.5 cells treated with 5 µM GCA (Fig. 7F). This 5 µM GCA-induced reduction in NS1 secretion appears to be independent of changes to Golgi morphology, as no apparent differences in NS1 and the Golgi marker GM130 staining patterns were observed by confocal immunofluorescence microscopy, and co-localization analysis indicated no significant impact on NS1 co-localization with GM130 (Fig. 7G). Collectively, these findings indicate that, when GCA is applied at 24 hours post-infection, the catalytic activity of GBF1 is dispensable for DENV genome replication but is required for efficient virus secretion and NS1 secretion.

## DISCUSSION

To identify host proteins that are involved in the secretion of DENV NS1, we performed a customized membrane-trafficking siRNA screen in human Huh-7.5 hepatoma cells infected with the DENV2-NS1-NLuc reporter virus. Our screen revealed the coatomer protein complex I (COPI) subunits COPA, COPB2, and COPG1, as the top three hits whose depletion reduced extracellular levels of NS1, thus heavily implicating COPI as an important determinant of DENV NS1 secretion. COPI has recently been identified as being involved in various aspects of DENV biology (50, 51); however, our study is the first to directly implicate COPI as an important determinant of DENV NS1 secretion.

Consistent with the results of our screen, previous studies have also identified several of our other top-ranking hits as host genes that are involved in orthoflavivirus NS1 biology. Intriguingly, dynamin-1 (DNM1) and early endosome antigen 1 (EEA1) are involved in the internalization of sNS1 (52), suggesting that these proteins may be involved in the bidirectional trafficking of secretion-destined NS1 and internalized sNS1. Similarly, valosin-containing protein (VCP) has recently been shown to co-localize with NS1 protein in cells infected with Japanese encephalitis virus (53), raising the possibility that VCP-NS1 interactions may be a feature shared among orthoflaviviruses. The identification here of vav guanine nucleotide exchange factor 2 (VAV2) as a potential determinant of NS1 secretion may also relate to the reported involvement of VAV2 in DENV-induced inflammatory responses (54). As the COPI components COPA, COPB2, and COPG1 were identified as the strongest hits in the siRNA screens, we focused on them and the COPI vesicle regulator GBF1 in subsequent validation and mechanistic studies.

COPI is a highly conserved protein complex that coats transport vesicles that shuttle protein and lipid cargo between cellular compartments. The complex consists of seven coatomer subunits ($\alpha ,\beta ,\beta^{`},\delta ,\varepsilon ,\gamma ,\zeta )$)(55). Mechanistically, COPI vesicle formation requires GBF1-catalyzed hydrolysis of GDP for GTP on ADP-ribosylation factors (Arf) (35, 56). Activated Arfs then recruit preassembled cytosolic heptameric COPI complexes to a donor membrane (57). The continued recruitment of COPI complexes to the nascent vesicle results in membrane destabilization and ultimately culminates in vesicle scission (29). The newly formed COPI-coated vesicle, complete with membrane-bound and luminal cargo, is then disseminated to its target acceptor membrane location (58). The best-categorized role of COPI-coated vesicles is their involvement in the bidirectional trafficking of proteins and lipids within the early secretory pathway (30). COPI-coated vesicles function in intra-Golgi trafficking, mediating anterograde and retrograde transport (59, 60). They also mediate Golgi-to-ER recycling of escaped ER-resident proteins, thus maintaining the structural and functional integrity of these organelles (61). Several studies have implicated COPI components as performing a role in endosomal transport and function (62–65). More recently, COPI has been demonstrated to perform roles in a wealth of processes, including lipid metabolism (66), autophagy (67), mRNA localization (68), nuclear envelope disassembly (69), and neurogenesis (70, 71). Regarding COPI vesicle regulators, GBF1 is well documented as being involved in multiple aspects of orthoflavivirus replication (reviewed in reference [44]). In addition, several Arfs have also been shown to play overlapping and redundant roles in DENV biology (50, 72). While these components regulate COPI vesicle formation, it must be noted that they have multiple effectors (73–75). However, given that the effects of GBF1 and Arf inhibition on orthoflavivirus biology can be phenocopied by COPI component depletion (50), this strongly suggests that COPI is involved in multiple aspects of the orthoflavivirus life cycle. Crucially, a recent study by Iglesias et al. (50) demonstrated that DENV utilizes COPI for the trafficking of capsid protein between the ER and lipid droplets, highlighting that the exploitation of COPI machinery by DENV is not limited to the canonical role of COPI in the secretory pathway.

Given the diverse roles of COPI and its regulators in orthoflavivirus biology, to focus specifically on NS1 secretion while minimizing pleiotropic effects, we concentrated our attention towards perturbing the COPI pathway at later stages of infection. Using this approach, we confirmed that RNAi-mediated depletion of COPI did not significantly impact intracellular DENV viral RNA replication or infectious virus production. Importantly, however, COPI depletion did result in a decrease in extracellular levels of NS1, coincident with an increase in the intracellular levels of NS1 for both DENV and WNV/KUNV, indicating that COPI siRNA-mediated depletion impairs the efficient secretion of NS1 for multiple orthoflaviviruses. While the modest levels of NS1 secretion inhibition observed here may reflect incomplete COPI protein knockdown, as preassembled heptameric COPI complexes are relatively stable and display a half-life of ~28 h in mammalian cells (76), these results also suggest the possible existence of multiple mechanisms that may be exploited by orthoflaviviruses to achieve NS1 secretion from human cells.

Intriguingly, pathogenic COPI SNP variants have been implicated in causing disease phenotypes that reflect those associated with orthoflavivirus complications, including arthritis (39), hemorrhage (39), microcephaly (40, 77), and dysregulation of the immune system (41). By overexpressing wild-type or deleterious COPI alleles in cells co-transfected with a replication-independent DENV non-structural protein expression vector, we were able to assess the effect of COPI perturbation on NS1 secretion independently from genome replication and infectious virus spread. The increase in the secretion efficiency of NS1 from cells transfected with the wild-type COPB2 construct suggests that the availability of COPB2 protein, in particular, may be a limiting factor in NS1 secretion. However, this conclusion is not entirely consistent with the comparable effects of RNAi-mediated knockdown of all three of these COPI components, and further studies are required to determine whether any one of these components is more important for NS1 secretion than the others. Unexpectedly, the overexpression of the dominant-negative mutant COPA^-E241K^ appeared to enhance NS1 secretion. This COPA allele contains a mutation in the WD40 domain that disrupts the binding of COPA to proteins that are otherwise targeted for Golgi-to-ER retrograde trafficking and leads to increases in ER stress (39). Whether the observed increase in NS1 secretion is a direct effect of COPA^-E241K^ expression remains an open question. It is possible that NS1 secretion is achieved via a non-canonical COPI function whereby the WD40 domain of COPA may be dispensable or even inhibitory to NS1 secretion. Alternatively, NS1 secretion may be favored under conditions of enhanced ER stress brought about by COPA^-E241K^ overexpression. It was also noteworthy that colocalization of NS1 with COPA^-E241K^ was significantly lower than that of wild-type COPA. If COPI vesicles play an indirect role in NS1 secretion, for example, via modulation of lipid homeostasis and lipid droplet budding processes that are important for formation of secretion-destined and lipid-associated higher-order NS1 oligomers, it is possible that COPA^-E241K^ expression may alter lipid homeostasis in a manner that favors NS1 secretion but contrasts effects of COPA knockdown. Nonetheless, how the overexpression of the COPA^-E241K^ variant enhances NS1 secretion warrants further investigation. Similar to the effects of COPA^-E241K^, COPB2^-R254C^, and COPG1^-K652E^ also show defects in Golgi-to-ER trafficking and aberrant cellular responses (40, 41, 78). Specifically, the COPB2^-R254C^ mutation is located within its N-terminal WD40 repeat domain, which is involved in binding to COPI cargo (79), and it has been hypothesized that COPB2 binds a specific subset of COPI cargo and that this binding may be incompletely perturbed by the R254C mutation (40). Similarly, the COPG1^-K652E^ mutation has been shown to disrupt the binding of COPI to the KDEL receptor and hamper the retrieval from the Golgi to the ER of chaperones that contain the KDEL motif (41). COPB2^-R254C^ and COPG1^-K652E^ are, however, not dominant-negative, and as such, their heterologous expression in a wild-type COPI background may have masked any potential effect on NS1 secretion. Despite our apparent inability to generate COPI component knockout cell lines by CRISPR/Cas9 technology, the use of genome editing to introduce these genetic variants in the place of wild-type genes offers an attractive approach to further explore the emerging roles of COPI in orthoflavivirus biology.

To functionally inhibit the formation of COPI vesicles, we employed the small molecule inhibitor Golgicide A (GCA). While it is well documented that GCA mediates a variety of effects on orthoflavivirus biology (44), most studies have utilized this compound and BFA at early timepoints during infection. Importantly, time of addition studies have shown that these compounds influence multiple aspects of the orthoflavivirus life cycle. When applied to WNV/KUNV-infected cells during the 12–16 h latent phase of infection (45), BFA inhibits the formation of virus-induced membrane structures (46) and severely impairs viral protein production and infectious virus release (47). However, when added late in infection, the virus-induced membrane structures are relatively stable (46), and only minor effects on protein synthesis were observed (47). Comparatively, GCA pulse-chase studies in DENV-infected cells indicate that despite having no impact on DENV internalization, intracellular viral RNA abundance is significantly reduced when GCA is applied in the first 12 h of infection, reduced to a lesser extent when applied at 12 h.p.i., but unaffected when applied at 24 h.p.i. (48). Indeed, the extracellular accumulation of orthoflavivirus NS1 is decreased in orthoflavivirus-infected mammalian cells when treated with high concentrations of BFA or GCA at 1 h.p.i. (49). However, given the inhibitory effects of these compounds on viral RNA replication, protein synthesis, and infectious virus production when administered early during infection, this is not surprising. As such, to mitigate the inhibitory effect of GCA on DENV RNA replication, we examined the impact of GCA treatment on NS1 secretion in DENV-infected cells when GCA is administered late in infection. Specifically, we functionally inhibited GBF1 using GCA at a later stage of infection and found that GCA reduces infectious DENV production in a dose-dependent manner. Consistent with a GCA-mediated defect in infectious virus production, we observed a concomitant increase in the intracellular levels of DENV viral RNA. The use of a DENV subgenomic replicon verified that GCA was not impacting viral RNA genome replication, confirming that GCA acts to prevent the release of infectious DENV virions. Interestingly, while virion secretion was reduced by GCA treatment in a dose-dependent manner, NS1 secretion was only observed to be reduced at the highest dose applied. In this context, it is noteworthy that our studies demonstrated significant inhibition of infectious virus production following GBF1 inhibition via GCA treatment but no significant impact of siRNA-mediated GBF1 knockdown on infectious virus production. While GCA treatment may be expected to result in a more complete inhibition of GBF1 activity in every cell, as compared to heterogeneous and incomplete knockdown of GBF1 expression via siRNA transfection, it is also possible that the discrepancies that we observed between the effects of GBF1 inhibition and knockdown on virus production reflect genuine differences in the biological consequences of these treatments and/or unanticipated off-target effects of one of these treatments. Future experiments involving analysis of the sensitivity of virus production to GCA following complete knockdown or knockout of GBF1 could help to resolve these discrepancies. Importantly, our results indicate that infectious DENV production is more sensitive than NS1 secretion to the effects of GCA-mediated GBF1 inhibition. These results provide further support for the conclusion that multiple mechanisms, both GBF1-dependent and GBF1-independent, may be exploited by orthoflaviviruses to achieve NS1 secretion from human cells.

**Fig 8:**
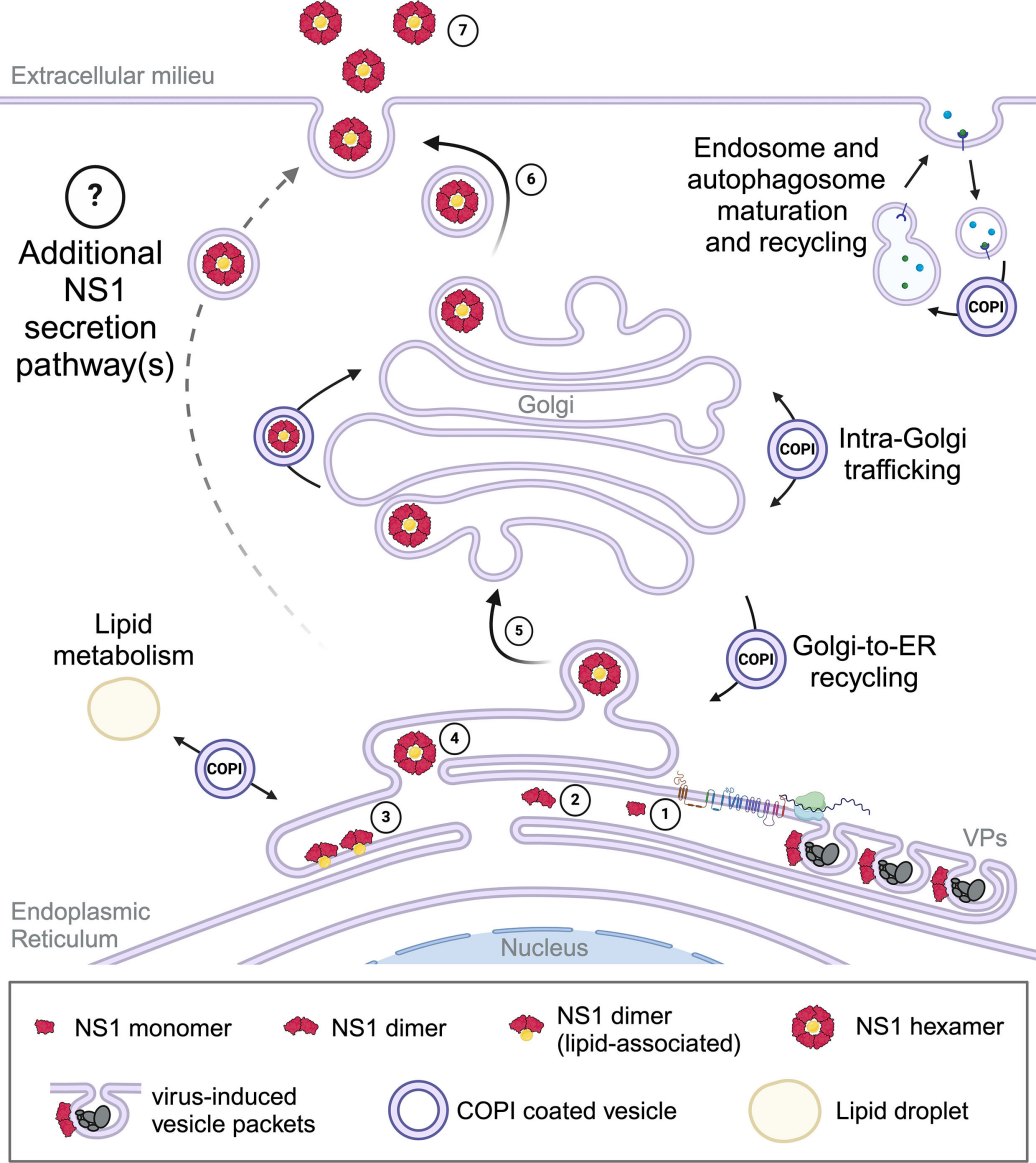
Potential sites of COPI involvement in DENV NS1 secretion (1). DENV NS1 is translated into the ER as a soluble monomer and modified by the addition of high-mannose glycans at N130 and N207 (2). Soluble monomers rapidly homodimerize to form a partially hydrophobic and membrane-associated dimer, the predominant intracellular NS1 form that plays a critical role in viral genome replication (vesicle packet (VPs) (3). Membrane-associated NS1 dimers are proposed to concentrate at sites of nascent lipid droplets within the ER or cholesterol-rich microdomains within the Golgi (4). Three membrane-associated dimers come together and pinch off from the membrane to form a soluble NS1 hexamer that is stabilized by a central lipid component (5). Secretion-destined NS1 is proposed to traffic from the ER to the Golgi for additional processing of the N130 moiety to a complex-type glycan (6). NS1 is proposed to be dispatched from the trans-Golgi network to the plasma membrane, where it is released into the extracellular environment (7). Secreted NS1 promotes viral propagation and contributes to dengue disease pathogenesis through a variety of pathways. Potential sites of COPI participation in NS1 secretion are shown (see Discussion). Created with BioRender.

Many genetic, biochemical, and imaging studies have been performed to interrogate NS1 secretion biology, and these have been integral in defining sNS1 structure and key functional residues. It is widely assumed that DENV NS1 is secreted from mammalian cells via the canonical secretion pathway (80). Multiple studies have indicated that DENV NS1 is translated into the ER as a soluble monomer and decorated with high-mannose moieties at N130 and N207 (6, 7, 81, 82). NS1 monomers rapidly homodimerize to form a partially hydrophobic NS1 dimer, the predominant intracellular form (6, 7). Membrane-associated NS1 dimers are proposed to preferentially localize to the sites of nascent lipid droplets on the luminal side of the ER (13), or to cholesterol-rich microdomains within the Golgi apparatus (3, 5, 12, 13). This has been suggested as a mechanism to concentrate NS1 dimers, with three dimers thought to come together to pinch off from the membrane, converting them into a soluble hexamer and collecting the lipid component that fills the hexamer’s central cavity (13). While not a strict prerequisite for NS1 secretion (82–84), the secreted form of NS1 exhibits a complex-type glycan at N130 (82). It is proposed that this additional processing of the N130 glycan occurs following ER-to-Golgi translocation given that, in uninfected cells, the machinery responsible for this maturation resides in the Golgi (7). Secretion-destined NS1 is understood to then traffic from the trans-Golgi network to the plasma membrane, where it exits the cell as a hexameric glycolipoprotein (80, 85). This study confirms that COPI components are important determinants of NS1 secretion, and this is compatible with the canonical secretion pathway model. However, the results of our GCA experiments are particularly intriguing. It is well established that DENV virions are matured as they traffic through the secretory pathway prior to being released from the cell as fully infectious virions (86). While the relatively low concentrations of GCA employed here revealed a dose-dependent reduction in infectious DENV production, a reduction in NS1 secretion was only observed at the highest dose (5 µM). Moreover, despite a dramatic decrease in NS1 secretion in cells treated with 5 µM GCA, our confocal microscopy studies showed no significant impact of GCA on NS1 and Golgi marker GM130 co-localization. Given the additional and emerging roles of COPI beyond intra-Golgi and Golgi-to-ER trafficking, and the demonstration that DENV exploits a non-canonical role of COPI to traffic capsid protein, alternative roles of COPI involvement in NS1 secretion warrant consideration. Potential sites of COPI involvement in NS1 secretion are shown in Fig. 8. Future studies interrogating the contribution that COPI-coated vesicles, their activators, and their vesicle constituents play in orthoflavivirus NS1 secretion will be integral to defining the role(s) of COPI in NS1 secretion and may provide additional targets for NS1-specific anti-orthoflaviviral therapies.

## MATERIALS AND METHODS

### Cell culture

Huh-7.5 cells (87) were generously provided by Charles M. Rice (Rockefeller University, New York, USA). Huh-7.5+FLuc cells stably expressing firefly luciferase have been previously described (88). Huh-7.5+T7 RNApol cells stably expressing T7 RNA polymerase have been previously described (89). All cells were maintained as previously described (89).

### Antibodies and chemicals

Mouse anti-NS1 monoclonal antibody (MAb) 4G4 was generously provided by Jody Peters and Roy Hall (University of Queensland, Brisbane, Australia) (90) or purchased from Mozzy Mabs (University of Queensland, Brisbane, Australia). Mouse anti-capsid MAb 6F3.1 was kindly provided by John Aaskov (Queensland University of Technology, Brisbane, Australia) (91). The mouse anti-Envelope monoclonal antibody was prepared from the hybridoma cell line D1-4G2-4-15 (4G2) purchased from ATCC and maintained as previously described (92). Mouse anti-β-actin MAb (AC-15) was purchased from Sigma-Aldrich. Mouse anti-COPA MAb (sc-398099) was purchased from Santa Cruz Biotechnology. Rabbit anti-COPB2 polyclonal antibody (PAb) (ab2899) and rabbit anti-GBF1 PAb (ab86071) were purchased from Abcam. Rabbit anti-COPG1 PAb (PA5-65194) and rabbit anti-GFP PAb (A-11122) were purchased from Thermo Fisher Scientific. Rabbit anti-GM130 MAb (D6B1) was purchased from Cell Signaling Technology. Alexa Fluor 488- and 555-conjugated anti-mouse IgG and anti-rabbit IgG secondary antibodies were purchased from Thermo Fisher Scientific. Fluorescent stain 4′,6-diamidino-2-phenylindole (DAPI) was purchased from Sigma-Aldrich. Revert 700 Total Protein Stain and IRDye 800 CW goat anti-mouse secondary antibody for Western Immunoblotting were purchased from LI-COR Biosciences. Golgicide A was purchased from Sigma-Aldrich and dissolved in dimethyl sulfoxide (DMSO) to 10 mM, aliquoted, and stored at −20°C.

### Virus plasmids and virus propagation

Plasmid pFK-DVs containing a full-length DENV2 genome (strain 16681), a pFK-DVs subgenomic replicon derivative encoding a *Renilla* luciferase construct (pFK-sgDVs-R2A), a pFK-sgDVs-R2A replication-deficient NS5 mutant derivative (pFK-sgDVs-GND-R2A), and a replication-independent T7-driven DENV2 non-structural protein 1-5 expression system (pIRO-D) was generously provided by Ralf Bartenschlager (University of Heidelberg, Heidelberg, Germany) (42, 93). pFK-DVs-NS1-NLuc bearing a NanoLuc luciferase tag within NS1 was created as previously described (8). Infectious DENV2 stocks were generated following *in vitro* transcription of DENV2 RNA using mMessage mMachine SP6 RNA polymerase (Thermo Fisher Scientific) using *Xba*I-linearized plasmid as template DNA and transfection of Huh-7.5 cells with purified RNA using DMRIE-C (Thermo Fisher Scientific) transfection reagent, as previously described (92). WNV/KUNV was generously provided by Karla J. Helbig (La Trobe University, Melbourne, Australia).

### Generating GFP-tagged wild-type and SNP variant COPI cDNA expression constructs and associated stable cell lines

To generate GFP-tagged wild-type COPB2 and COPG1 cDNA expression constructs, FLAG-tagged COPB2 and COPG1 cDNA ORF clone plasmids (GenScript: Cat No. OHu04585 and Cat No. OHu29546, respectively) served as cDNA templates. For COPA cDNA, total cellular RNA was isolated from Huh-7.5 cells using NucleoZOL (Macherey-Nagel), as per the manufacturer’s instructions. First-strand cDNA was synthesized using M-MLV Reverse Transcriptase (Promega) in conjunction with random hexamer primers as per manufacturer’s instructions. COPA, COPB2, and COPG1 cDNA were PCR amplified using gene-specific primers and Q5 High-Fidelity DNA Polymerase (New England Biolabs). Gel-purified (Macherey-Nagel) amplicons were assembled as in-frame fusions with emGFP PCR product into a pLenti6/V5-D-TOPO vector using NEBuilder HiFi DNA Assembly (New England Biolabs). cDNA sequences were confirmed by Sanger Sequencing (Australian Genome Research Facility, Adelaide, Australia).

For SNP incorporation, GFP-tagged COPI cDNA constructs were modified by QuikChange II site-directed mutagenesis (Agilent) in conjunction with SNP-specific primers and confirmed by Sanger sequencing. Exact cloning details for these constructs are available on request (Table S2).

To generate stable Huh-7.5 cell lines expressing emGFP-tagged COPA, COPB2, and COPG1, the respective pLenti6/V5-D-TOPO plasmids containing these cassettes were packaged into lentiviral vectors and used to transduce Huh-7.5 target cells as described previously (94). Following selection with 5 µg/mL of Blasticidin HCl (Thermo Fisher Scientific) for approximately 2 weeks, emGFP-positive cells were sorted using a BD FACSAria III Fusion Flow Cytometer (BD Biosciences) at the Flinders University Flow Cytometry Facility and expanded and maintained as polyclonal cell lines.

### Generation of lentiviral shRNA expression vectors and shRNA cell lines

The TRIPZ Inducible Lentiviral Non-silencing shRNA Control (pTRIPZ-shNTC) plasmid was purchased from Open Biosystems (Horizon Discovery). Derivative plasmids encoding two different top-ranking microRNA-adapted shRNAs for each target gene (COPA [NM_001098398.2], COPB2 [NM_004766.3], and COPG1 [NM_016128.4]) were designed using the online tool “shRNAI” (http://big2.hanyang.ac.kr/shRNAI) (95). Specifically, pTRIPZ-shNTC was digested with *Xho*I and *Mlu*I-HF (New England Biolabs) to liberate the ~345 bp shNTC cassette, and the linearized ~13 kb plasmid backbone was gel purified (Macherey-Nagel) and individually assembled with synthetic DNA fragments (GenTitan Gene Fragments; GenScript) for the corresponding COPA, COPB2, and COPG1 shRNA-encoding cassettes using NEBuilder HiFi DNA Assembly (New England Biolabs). Following transformation into NEB 5-alpha Competent *E. coli* (High Efficiency) (New England Biolabs), individual plasmid DNA clones were prepared and confirmed by Sanger sequencing (Australian Genome Research Facility, Adelaide, Australia). Plasmids were then packaged into lentiviral vectors and used to transduce Huh-7.5 target cells, as described previously (94). Following selection with 3 µg/mL of puromycin dihydrochloride (Sigma-Aldrich) for approximately 3 weeks, polyclonal puromycin-resistant cell lines were used for qRT-PCR and Western blotting experiments, as indicated.

### siRNA library screening

The siRNA library comprised a commercially available library targeting membrane trafficking proteins (Human ON-TARGETplus siRNA Library—Membrane trafficking—SMARTpool, Dharmacon cat# G-105500) and 37 additional siRNA SMARTpools (Dharmacon, Horizon Discovery) targeting previously identified proviral host factors that may be manipulated by NS1 (33, 34). Each siRNA SMARTpool consists of four siRNAs targeting the same gene. A scrambled non-targeting control (NTC) siRNA served as a negative control. siRNAs targeting Firefly luciferase (FLuc) and NanoLuc luciferase (NLuc) served as controls for cell viability and inhibition of DENV replication, respectively. The siRNA library was prepared at 1 µM in siRNA buffer (Dharmacon cat# B-002000-UB-100), pre-arrayed in 96-well plates at a volume of 4 μL/well, as appropriate for individual screening experiments and stored at −80°C until further use. siRNA screening and screen data analysis were performed at Cell Screen SA (Flinders Centre for Innovation in Cancer, Flinders University, Australia).

For a schematic representation of the experimental setup, see Fig. 1B. Huh-7.5+FLuc cells were seeded into 75 cm^2^ flasks at 1.56 × 10^6^ cells/flask. The following day, cells were transfected with DENV2-NS1-NLuc *in vitro* transcribed (IVT) RNA using DMRIE-C for 3 h before the transfection reagent was replaced with complete media. At 48 hours post-transfection (h.p.t.), DENV2-NS1-NLuc RNA-transfected cells were trypsinized and reverse transfected with the siRNA SMARTpool library at a final concentration of 40 nM in 96-well plates. For this, prearrayed 96-well plates containing 4 μL of 1 μM siRNA SMARTPool siRNAs were incubated with 15.7 μL OptiMEM and 0.3 μL DharmaFECT4 (Horizon Discovery) for 20 min before DENV2-NS1-NLuc transfected cells were added at 1.25 × 10^4^ cells/well/80 μL. After 3 hours of incubation, siRNA transfection reagent-containing media was replaced with complete media (100 μL/well). For each experiment, each siRNA SMARTPool was transfected in triplicate, and three independent experimental replicates were performed. At 48 hours post-siRNA transfection, cell culture supernatants were collected, clarified by centrifugation (500 × *g*, 5 min, at 15°C), and mixed 1:1 with 2× passive lysis buffer (Promega cat #E1941). Cell monolayers were washed in PBS and lysed in 1× passive lysis buffer. Cell lysates and lysed supernatants were stored at −20°C until further use. To assess the impact of siRNA SMARTpool treatment on intracellular and extracellular NS1 abundance, samples were assayed using the Nano-Glo Dual-luciferase reporter (NanoDLR) assay (Promega), according to the manufacturer’s recommendations. Cell lysate or lysed supernatant was mixed with OneGlo reagent (Promega) and incubated for 45 min at RT prior to FLuc (cell viability) luminescence quantification using a PerkinElmer Ensight plate reader. Following FLuc luminescence measurements, NanoDLR Stop&Go reagent was added, mixed, and incubated for 45 min at room temperature (RT) prior to NS1-associated NLuc luminescence quantification. NLuc-associated luminescence values and FLuc-associated luminescence values for each siRNA treatment were expressed as a percentage of the corresponding average NTC siRNA-associated values. Means, S.D., and % CV were calculated on these normalized values and the test siRNA were scored to identify hits as detailed in the supplemental material.

A deconvolution screen was performed on the eight criteria-matching hits and GBF1. Here, each of the four individual siRNA duplexes comprising each siRNA SMARTPool was assayed in triplicate. In this screen, cell viability was additionally measured using CellTiter-Blue (Promega), as per the manufacturer’s instructions, using a PerkinElmer Ensight plate reader. Transfections and luciferase assays were performed as described above. Data analysis and hit scoring were performed as described above and in the supplemental material.

### siRNA treatment of flavivirus-infected cells

Huh-7.5 cells were grown in 75 cm^2^ flasks (5 × 10^6^ cells/flask) and cultured overnight. The following day, cells were infected with DENV2 or KUNV (MOI ~1) or mock infected. At 4 hours post-infection (h.p.i.), cells were trypsinized and reverse transfected with COPI siRNA SMARTpools at a final concentration of 40 nM in 12-well plates. For this, a transfection mix comprised of 2 µL of 20 µM siRNA SMARTPool, 195 µL OptiMEM, and 3 µL Dharmafect4 was incubated at RT for 20 min before DENV- or KUNV-infected cells were added at 1.8 × 10^5^ cells/well/800 µL. After 3 hours of incubation, siRNA transfection reagent-containing media was replaced with complete media (1 mL/well). At 24 h.p.i., cells were washed and media was replaced with complete DMEM (400 µL/well). At 48 h.p.i., cell culture lysates and supernatants were recovered to measure intracellular and extracellular NS1 abundance, respectively. In parallel, DENV-infected plates, total cellular RNA, and cell culture supernatants were collected to measure viral RNA levels, host RNA expression, and infectious virus production, respectively.

### COPI cDNA and pIRO-D expression vector co-transfection

Huh-7.5+T7 RNApol cells were seeded in 12-well plates at 1.2 × 10^5^ cells/well and cultured overnight in complete DMEM without puromycin. The following day, cells were co-transfected with 0.5 µg of each plasmid pIRO-D and COPI cDNA well using Lipofectamine 3000 (Thermo Fisher Scientific) according to the manufacturer’s instructions, and transfection reagent-containing media was replaced with complete DMEM at 3 h.p.t.. At 18 h.p.t., cell culture lysates and supernatants were collected for Western blot analysis.

### Golgicide A (GCA) treatment of flavivirus-infected cells

Huh-7.5 cells were seeded into 75 cm^2^ flasks at 5 × 10^6^ cells/flask and cultured overnight. The following day, cells were infected with DENV2 or KUNV (MOI: ~1) or mock infected. At 4 h.p.i., cells were washed, trypsinized, and re-seeded into 12-well plates at 1 × 10^5^ cells/well for Western blotting, qRT-PCR or focus forming assays (FFA), or 96-well plates at 1 × 10^4^ cells/well for cell viability assays and returned to culture in complete DMEM. At 24 h.p.i., cells were washed and cultured in complete DMEM supplemented with the indicated concentrations of GCA or DMSO carrier control, as indicated. At 18 hours post-GCA treatment, samples were harvested for Western blot, RT-qPCR, FFA, and cell viability analysis, as indicated.

### Quantification of mRNA and viral RNA by RT-qPCR

Total cellular RNA was extracted from near-confluent cells in 12-well plates using NucleoZOL (Macherey-Nagel), according to the manufacturer’s instructions. Both first-strand complementary DNA (cDNA) synthesis and RT-qPCR were performed using the Luna Universal One-Step RT-qPCR Kit (New England Biolabs) in 384-well plates using a QuantStudio 7 Flex (Life Technologies) or CFX Opus (Bio-Rad) thermal cycler according to the manufacturer’s recommendations. For each sample and each primer pair, 10 µL of reactions was prepared in technical duplicate, each containing 5 ng of total RNA, 0.2 µL of each primer at 20 µM (0.4 µM final concentration), 0.5 µL Luna WarmStart RT Enzyme Mix, and DNase/RNase-Free water. Melt curve analysis was performed using the qPCR instrument default settings. mRNA or viral RNA levels were expressed as a percentage of the experimental control (NTC or DMSO, as appropriate) following normalization to RPLPO mRNA, using the threshold cycle (ΔΔCT) method. Primer sequences are available upon request.

### Quantification of subgenomic DENV RNA replication by luciferase assay

Subgenomic RLuc-encoding DENV2 replicons were subjected to *in vitro* RNA transcription as previously described (92). Huh-7.5 cells were seeded in 12-well plates at 1 × 10^5^ cells/well and cultured overnight. The following day, cells were transfected with IVT RNA using DMRIE-C (Thermo Fisher Scientific) according to the manufacturer’s instructions. At 3 h.p.t., transfection reagent-containing media was replaced, and cells were returned to culture in complete DMEM. At 24 h.p.t., cells were washed and returned to culture in complete DMEM supplemented with increasing concentrations of GCA or 0.1% (vol/vol) DMSO carrier control alone, as indicated. At 18 hours post-GCA treatment, cell culture monolayers were lysed in Renilla Luciferase Lysis Buffer (Promega). Samples were mixed with Renilla Luciferase Assay Reagent (Promega) according to the manufacturer’s instructions, and luminescence was determined using a Cytation 5 Multimode Reader equipped with a Dual-Reagent Injector Module (BioTek).

### Quantification of protein knockdown by indirect immunofluorescence microscopy

Huh-7.5 cells were reverse transfected in 12-well plates at 1 × 10^5^ cells/well with COPI or NTC siRNA SMARTpools at a final concentration of 40 nM. At 3 h.p.t., transfection media was replaced with complete DMEM, and cells were returned to culture. At 24 h.p.t., cells were trypsinized and re-seeded into 96-well black-walled imaging plate (PerkinElmer PhenoPlate-96) at 1 × 10^4^ cells/well and returned to culture. At 48 h.p.t., cells were washed with PBS prior to fixation using ice-cold acetone:methanol (1:1), for 5 min at 4°C and then blocked using 5% (wt/vol) BSA in PBS for 30 min at RT. Samples were labeled with indicated primary antibodies anti-COPA (1:50), anti-COPB2 (1:100), anti-COPG1 (1:100), or anti-GBF1 (1:100) diluted in 1% BSA in PBS at 4°C overnight. Samples were then washed three times in PBS before incubation for 2 h in the dark at 4°C with Alexa Fluor 488-conjugated anti-rabbit IgG or Alexa Fluor 488-conjugated anti-mouse IgG antibodies (Thermo Fisher Scientific), as appropriate, diluted to 1:500 and 1:2,000, respectively. Samples were then washed three times in PBS and counterstained with DAPI (Sigma-Aldrich) diluted to 1 µg/mL in PBS for 10 min in the dark at RT before being washed in PBS. Samples were then imaged using a BioTek Cytation 5 Multimode Reader. Wells were imaged using a 10× objective across a 7 × 7 montage. The images were processed and analyzed using BioTek Gen5 software (version 3.08.01). Briefly, cellular analysis was performed by defining individual cells using a primary mask based on DAPI fluorescence and an appropriate object selection size (10 µm–50 µm) and a secondary mask expanding from the DAPI-defined nuclear membrane by 30 µm. The sum intensity of COPI labeling-associated green fluorescence within the secondary mask was measured for each cell as a measure of protein abundance.

### Cell viability assays

Cell viability assays were performed using a CellTiter-Glo 2.0 luminescent cell viability assay (Promega) as per the manufacturer’s instructions using a BioTek Cytation 5 Multimode Reader.

### Infectivity assays

Virus-containing cell culture supernatants were recovered from indicated experiments at the specified timepoints, clarified by centrifugation, and stored at −80°C. Infectivity was assessed by focus forming assay (FFA), as previously described (8).

### SDS-PAGE and western blotting

Cell culture supernatants and lysates were recovered from infected and transfected Huh-7.5 cells and derivatives as detailed above. Supernatants were clarified by centrifugation (16,000 × *g* for 5 min at 4°C), divided into aliquots, mixed with non-reducing sample buffer, boiled at 95°C for 5 min, and stored at −20°C. Lysates were harvested using NP40 lysis buffer (1% [vol/vol] NP-40, 50 mM Tris-HCl [pH 8.0], 150 mM NaCl) containing protease inhibitor cocktail (Sigma-Aldrich), as described previously (89), divided into aliquots, mixed with reducing (for β-actin analysis) or non-reducing (for NS1 analysis) buffer, boiled at 95°C for 5 min, and stored at −20°C. Samples were separated by SDS-PAGE and transferred to nitrocellulose membranes (Bio-Rad). For cell culture supernatant normalization, total protein stain analysis was performed using Revert 700 Total Protein Stain (LI-COR), following the manufacturer’s instructions and imaged using a LI-COR Odyssey imaging system (Flinders Proteomics Facility, Flinders University, Australia). Following blocking (5% [wt/vol] skim milk in TBS for 1 h), membranes were incubated with primary antibody (anti-NS1 [1:10] or anti-β-actin [1:5,000] in TBS supplemented with 0.05% [vol/vol] Tween-20 [Sigma-Aldrich] and 1% [wt/vol] skim milk) at 4°C overnight. After stringent washing, membranes were incubated with IRDye 800 CW goat anti-mouse IgG secondary antibody (1:15,000) for 1 h in the dark at room temperature (RT). Membranes were then washed and imaged using a LI-COR Odyssey imaging system (Flinders Proteomics Facility, Flinders University, Australia), and signal intensities were quantified using Image Studio Lite (version 5.2.5). For quantitative analysis of NS1 protein secretion efficiency, intracellular NS1 (iNS1) signal intensities were first normalized to those of the loading control β-actin, and secreted NS1 (sNS1) signal intensities were normalized to those of the entire lane Total Protein Stain (TPS) values. Subsequently, normalized sNS1 values were divided by normalized iNS1 values and expressed as a percentage of that of the relevant negative control for that experiment.

### Immunofluorescent labeling and confocal microscopy

For GCA treatment experiments (Fig. 7), Huh-7.5 cells were infected with DENV (MOI ~1) in a 25 cm^2^ flask. At 4 h.p.i., cells were trypsinized and re-seeded at 1 × 10^4^ cells/well into #1.5 coverglass-bottomed µ-Slide 8-well chamber slides (ibidi Gmbh, Germany) that were pre-coated with 0.2% (wt/vol) gelatin and returned to culture for a further 18 h. Uninfected Huh-7.5 control cells were handled similarly in parallel. At 24 h.p.i., cells were washed twice in complete DMEM and returned to culture in complete DMEM supplemented with increasing concentrations of GCA or 0.1% (vol/vol) DMSO vehicle control for a further 18 h. At 18 hours post-GCA treatment, cells were washed prior to fixation using ice-cold acetone:methanol (1:1) for 5 min at 4°C. Cell monolayers were then washed with PBS and blocked using 5% (wt/vol) BSA in PBS for 30 min at RT. Samples were labeled with primary antibodies anti-NS1 (1:5) and anti-GM130 (1:1,000) diluted in 1% (wt/vol) BSA in PBS at 4°C overnight. Samples were then washed three times in PBS before incubation for 1 h in the dark at 4°C with AlexaFluor 488-conjugated anti-rabbit IgG and Alexa Fluor 555-conjugated anti-mouse IgG antibodies (Thermo Fisher Scientific) diluted to 1:500 and 1:1,000, respectively. Samples were then washed three times in PBS and counterstained with DAPI (Sigma-Aldrich) at 1 µg/mL in PBS for 10 min in the dark at RT before being washed with PBS.

For confocal analysis of NS1 localization with respect to GFP-tagged COPA, COPB2, and COPG1 (Fig. 4), samples were prepared and labeled as above, with the exceptions that cells were seeded directly into gelatin-coated µ-Slide 8-well chamber slides, cultured overnight, and infected with DENV2 (MOI ~0.1). After culturing for 24 h, cells were fixed and blocked, as above, before incubation for 1 h at RT in anti-NS1 (diluted 1:5) and anti-GFP (diluted 1:200) antibodies diluted in PBS containing 1% BSA (wt/vol). Cells were then washed with PBS, incubated with secondary antibodies (both diluted to 1:200), and washed, as described above. For confocal analysis of NS1 localization with respect to wild-type and SNP variants of GFP-tagged COPA, COPB2, and COPG1 in pIRO-D-transfected cells (Fig. 6), samples were grown on gelatin-coated #1.5 coverslips, transfected, fixed, and labeled, as above.

Samples were imaged using a ZEISS LSM 880 Fast Airyscan confocal fluorescence microscope system using a C-Plan-Apochromatic 63× (NA: 1.4) oil immersion objective (Flinders Microscopy and Microanalysis, Flinders University, Australia). For pIRO-D-transfected samples (Fig. 6) and GCA treatment samples (Fig. 7), laser lines 405 nm, 488 nm, and 561 nm were used at 2% maximal power with appropriate detector master gain settings to enable signal visualization with minimal saturation. Pinhole sizes were set to 1.0 Airy units for the longest-wavelength fluorophore and matched for all tracks. For confocal analysis of NS1 localization with respect to GFP-tagged COPA, COPB2, and COPG1 in DENV-2-infected cells (Fig. 4), the 32-channel Airyscan detector was used in combination with appropriate Main Beam Splitter, Second Beam Splitter, and Emission Dual Filters, the SR Airyscan Mode, and appropriately reduced Master Gain settings (<50% of the dynamic range) and laser powers to satisfy Airyscan automatic alignment requirements. For this, images were acquired using 3× zoom and Z-stack intervals of 0.15 µm and processed using 3D Airyscan Processing and automatic conditions. Images were processed and analyzed using ZEN Blue (version 3.2) software (ZEISS). Where indicated, colocalization analysis was performed by measurement of Pearson’s correlation coefficients for each cell (>30 cells/group) following drawing Bezier regions of interest around each cell using ZEN Blue. Where indicated, line profiles of 15 µm were drawn through typical NS1 foci, and fluorescence intensities for both channels at each point along the line were exported and graphed using GraphPad Prism version 10.2.2 software.

### Statistical analysis and software

GraphPad Prism version 10.2.2 was used for statistical analyses and graphing. Unless otherwise indicated, data are means ± S.D. Details for statistical tests are provided in the Figure Legends. Figures were compiled using Adobe Photoshop version 24.1.0 software.

## AFTER EPUB

*[This article was published on 21 August 2025 with errors in figure order and citations. The errors were updated in the current version, posted on 22 August 2025.]*

## SUPPLEMENTAL MATERIAL

Supplemental methods jvi.00962-25-s0001.pdf Additional experimental details.

Table S1 jvi.00962-25-s0002.xlsx Primary siRNA screen data (related to Fig. 1C).

Table S2 jvi.00962-25-s0003.xlsx Secondary ("deconvolution") siRNA screen data (related to Fig. 1D).

ASM does not own the copyrights to Supplemental Material that may be linked to, or accessed through, an article. The authors have granted ASM a non-exclusive, world-wide license to publish the Supplemental Material files. Please contact the corresponding author directly for reuse.
