# Supplementary material for: Coatomer protein complex I is required for efficient secretion of dengue virus non-structural protein 1
Source: J Virol. 2025 Aug 21;99(9):e00962-25. doi: 10.1128/jvi.00962-25 (PMC12455993; doi:10.1128/jvi.00962-25)
Supplement: Supplemental methods — Additional experimental details. [file jvi.00962-25-s0001.pdf]

# Methods: siRNA Screen Data Analysis

## Primary siRNA Screen (related to Figure 1C)

### Data structure and compilation

The luminescence readings after 45 min of incubation with the respective luciferase substrates were used to measure the enzymatic activity of NLuc and FLuc which is directly related to the relative quantities of both enzymes present in the samples. As described in the Materials and Methods, samples consisted of Passive Lysis Buffer (Promega)-inactivated supernatants and cell lysates collected after DENV transfection and siRNA mediated gene knockdown. Accordingly, every test siRNA- or control siRNA-transfected well generated 2 samples corresponding to lysates and supernatants and both NLuc and FLuc activities were measured in each sample, thus generating 4 data points per well (treatment). Accordingly, each plate generates one file containing the data of all wells within the plate for each luciferase reading. The table below summarises the luciferase readings and the variables names assigned to them:

| Enzyme | Sample | Variable name |
|---|---|---|
| Firefly luciferase | Lysate | FLuc_Lys |
| Firefly luciferase | Culture supernatant | FLuc_Sup |
| NanoLuc luciferase | Lysate | NLuc_Lys |
| NanoLuc luciferase | Culture supernatant | NLuc_Sup |

The data for all plates (all replicates) were exported as individual files (4 per each plate) from the Ensight multimode plate reader (Revvity) in a csv format. All data files were then compiled into one single database containing all readings from all plates using a Python script. During compilation the script also generated identifiers for all data points as for treatment (test siRNAs and controls), source sample, luciferase type, replicate number (within the plate) and experimental replicate number (plate replicate). This annotated database was then imported into Spotfire (TIBCO Spotfire Desktop 10.8.0) for further data processing and analysis.
The correctness of identifier assignment to data points was confirmed by crosschecking with the plate's layouts according to the experimental design using cross-tables, heatmaps and scatter plot visualisations in Spotfire.

### Data exploration and Quality Control:

The data was assessed for quality using general statistics for central tendency and variability on controls (non-targeting siRNA control, NT_siRNA) such as mean, standard deviation and coefficient of variation (%CV) on a per plate basis (4 NT_siRNA treated wells per plate). No significant differences were observed for control values across all plates, with the %CV remaining below 8%. Similarly, the %CV for all controls for each replica was below 10%.
A general exploration of the data was also carried out for the FLuc_Lys values on a per plate basis to assess variability and identify outliers. Outliers were identified using the inter-quartile range and upper and lower inner fences as boundaries and the corresponding data set (well) was removed from the analysis. After outlier removal, the %CV for FLuc_Lys values across all replicas remained below 15%.

FLuc activity in the lysates was not only used to assess consistency and variability but also for data normalization under the assumption that the knockdown of selected gene by siRNA transfection should not affect the FLuc expression. This was confirmed by comparing the average values of FLuc_Lys readings for every gene to the average values of the non-targeting

siRNA control of the corresponding plate. Only siRNA pool targeting the gene RHOA consistently reduced the levels of NLuc_Lys, so this gene was removed from the analysis.
Because FLuc secretion to the culture media was not expected but also not relevant for the purposes of the screen, the readings corresponding to FLuc activity in the supernatants were not considered in the data analysis although they were measured and rendered almost undetectable levels, as expected.

**Data Normalisation:**
For calculation and data normalisation, all wells were considered as independent treatments and the replicates were only averaged after normalisation.
The NLuc relative levels (RL) in lysates and supernatants were calculated as ratios of Fluc_Lys values in order to normalise for variations of the cell densities. In this case:

$$RL\_NLuc\_Lys = \frac{NLuc\_Lys}{FLuc\_Lys} \qquad RL\_NLuc\_Sup = \frac{NLuc\_Sup}{FLuc\_Lys}$$

The NLuc secretion ratio (SR) to the media was also calculated as a ration of supernatant to lysates readings:

$$SR\_Nluc = \frac{NLuc\_Sup}{NLuc\_Lys}$$

Following this, NLuc RL and SR values were normalised as percentages of average values of the NT_siRNA controls of corresponding plates.

$$Normal\_NLuc\_Lys = \frac{RL\_NLuc\_Lys\ (well)}{Mean\ RL\_NLuc\_Lys\ (NT\_siRNA)} * 100$$

$$Normal\_NLuc\_Sup = \frac{RL\_NLuc\_Sup\ (well)}{Mean\ RL\_NLuc\_Sup\ (NT\_siRNA)} * 100$$

$$Normal\_SR\_NLuc = \frac{SR\_NLuc\ (well)}{Mean\ SR\_NLuc\ (NT\_siRNA)} * 100$$

Normalised values were then used for further calculations and identification of hits.

**Identification of hits:**

Several factors must be considered for identifying genes (hits) involved in NS1 secretion, when measuring the secretion levels after transient siRNA-mediated mRNA knockdown. For hits identification, four effects were considered as possible for any test siRNA and thus contributing to the overall effect on NLuc secretion as a measure of NS1 protein expression and release:

1. Cell toxicity, indirectly measured as a significant decrease of FLuc activity: **FLuc knockdown**.
2. Inhibition of NLuc activity in lysates, measured as a significant decrease of *Normal_NLuc_Lys* values: **NLuc_Lys knockdown.**
3. Inhibition of NLuc activity in supernatants, measured as a significant decrease of *Normal_NLuc_Sup* values: **NLuc_Sup knockdown.**

4. Inhibition of NLuc secretion, measured as a significant decrease of *Normal_SR-NLuc* values: **Secretion knockdown.**

For determining the knockdown effects, thresholds were calculated for all possible effects individually and Boolean values (TRUE or FALSE) were assigned to every test siRNA using conditional functions to test if the values fell over the respective threshold.

All thresholds (one for each possible effect) were calculated based on the central tendency and variation of the normalised values of NT_siRNA controls. For this, control values from all replicates were averaged and the mean and standard deviation values were used for threshold calculation. The threshold for FLuc knockdown, NLuc_Sup knockdown and NLuc_Lys knockdown was set at one standard deviations below the mean value while for Secretion knockdown, a threshold of two standard deviations from the mean was used.

To assist with hits identification a scoring system was developed, assigning numerical values (scores) to each knockdown effect according to the significance (relevancy) for the experimental model. The score values were selected in a way that the rank resulting from the addition of all scores (total scores) would generate a unique value for all possible combinations of effects. Scores were assigned to treatments based on the Boolean values after conditional comparison with respective thresholds.

The scores for each effect are shown in the tables below:

**Treatment scoring system according to the detected effects**

| Effect | Parameter | Criteria | Threshold (calculated from NT_siRNA control) | Score | |
|---|---|---|---|---|---|
| | | | | TRUE | FALSE |
| *Secretion knockdown* | Normal_SR_NLuc | < | Mean SR_NLuc - 2SD | 8 | 0 |
| *NO FLuc knockdown* | FLuc | > | Mean FLuc - 1SD | 4 | 0 |
| *NLucLys knockdown* | Normal_NLuc_Lys | < | Mean Normal_NLuc_Lys - 1SD | 2 | 0 |
| *NLucSup knockdown* | Normal_NLuc_Sup | < | Mean Normal_NLuc_Sup - 1SD | 1 | 0 |

**Treatment classification based on the total scores**

| Treatment Classification | Total Score | Effects | | | |
|---|---|---|---|---|---|
| | | *Secretion knockdown* | *NO FLuc knockdown* | *NLuc_Lys knockdown* | *NLuc_Sup knockdown* |
| *HIT* | ≥ 12 | T | T | T/F | T/F |
| *Potential HIT* | 5 - 11 | F | T | T | T/F |
| *No Effect* | < 5 | F | T | F | F |

T: True, F: False, T/F: either True or False

Based on the total score, each gene (siRNA pool) was classified into Hit, Potential Hit or No Effect.

- Hit: total score ≥ 12, suggesting that the gene knockdown resulted in no effect on FLuc levels but knocked down the secretion of NLuc.

- Potential Hits: total score 5 - 11, suggesting that the gene knockdown did not affect the FLuc expression, knocked down the NLuc expression but had no effect on the secretion.
- No effect: total score < 5, suggesting no effect at all in any of the enzyme's levels or a significant decrease of FLuc basal expression levels that could be considered as a toxic effect reducing cell viability.

## Deconvolution siRNA Screen of Hits (related to Figure 1D)

Data handling and analysis of the deconvolution siRNA screen was very similar to that of the primary screen and thus only the main differences or additions are detailed below.

### Data structure and compilation

For the deconvolution hit follow up, identified hits and selected genes were assayed as pooled (4 siRNA per pool as evaluated in primary screen) and individual siRNA in triplicates while 6 replicas of the non-targeting siRNA (NT-siRNA) control were included in each plate. Two experimental replicas using identical plate layouts were used.
Sample preparation and luciferase measurements were performed as described previously, so data sets with similar structures were generated. However, to counteract the possibility of direct effects of a test siRNA on FLuc expression levels, an additional measurement for cell viability was introduced. In addition to the luciferase assays, CellTiter-Blue (CTB; Promega) viability assays were also performed (as described in Materials and Methods), in order to normalise the data to the number of cells in the well.
Individual csv files in list format for each assay and sample were exported from the Ensight and compiled into a single database containing the annotated data points from all plates. Compilation of all files was carried out using the same Python script with minor adjustment for additional data.
Further data handling and analysis was done using Spotfire.

### Data exploration and Quality Control:

The mean, standard deviation and %CV was calculated for the experimental replicates for each treatment on a per plate basis and used to assess data quality. In this case the central values and variability of the readings from CTB assays (viability) and RLU from FLuc were similar in the two experimental replicas and no significant effect was observed from any of the siRNAs on cell viability (toxicity) or FLuc expression. Similarly, outliers were detected on the control well and removed, resulting in %CV values lower than 10% for all plates and across the screen.

### Data Normalisation:

Considering that CTB assays are a more accurate measurement of cell viability, this data was used to calculate the relative expression values for both FLuc_Lys and NLuc as a point of difference from the normalisation method used in the primary screen, where FLuc_Lys values were used as a reference.
This normalisation method used the following formulas:

$$RE\_FLuc = \frac{FLuc\_Lys}{CTB\ Viability}$$

$$RE\_NLuc\_Lys = \frac{NLuc\_Lys}{CTB\ Viability}$$

$$RE\_NLuc\_Sup = \frac{NLucSup}{CTB\ Viability}$$

The NLuc secretion ratio (SR) to the media was also calculated as a ration of supernatant to lysate readings:

$$SR\_Nluc = \frac{NLuc\_Sup}{NLuc\_Lys}$$

Further normalisation was carried out using the same approach. Briefly, Normal_NLuc_Sup, Normal_NLuc_Lys and Normal_SR_NLuc were calculated as percentages of the mean of corresponding averages from NT_siRNA controls in every plate. The calculation was performed as described before.

**Hit identification:**
After normalisation, the methodology for Hits identification and siRNA effect classification was based on the same principles and followed the same procedure as described for the primary screening.

## Supplemental Material – Table Legends

**TABLE S1.** Primary siRNA screen data (related to Figure 1C).

**TABLE S2.** Secondary ('deconvolution') siRNA screen data (related to Figure 1D).
